# Comparative cost analysis of community-based tuberculosis screening: Computer-Aided Detection (CAD) alone versus CAD with point-of-care C-reactive protein testing in South Africa and Lesotho

**DOI:** 10.1371/journal.pgph.0007341

**Published:** 2026-09-21

**Authors:** Harsh Vivek Harkare, Anna Verjans, Phillip Joseph, Mashaete Kamele, Shriya Misra, Mamatlakeng Keitseng, Mojabeng Mabeleng, Thandanani Madonsela, Alastair van Heerden, Johanna Kurscheid, Bart K.M. Jacobs, Aita Signorell, Fiona Vanobberghen, Klaus Reither, Irene Ayakaka, Shannon Bosman, Fabrizio Tediosi

**Affiliations:** 1 Swiss Tropical and Public Health Institute, Allschwil, Switzerland; 2 University of Basel, Basel, Switzerland; 3 Epidemiology, Biostatistics, and Prevention Institute, University of Zurich, Zurich, Switzerland; 4 Centre d’Estudis Demogràfics (CED-CERCA), Universitat Autònoma de Barcelona, Barcelona, Spain; 5 Centre for Community Based Research, Human Sciences Research Council, Sweetwaters, South Africa; 6 SolidarMed, Butha Buthe, Lesotho; 7 SYNAPSE Research Collective, Wits Health Consortium, Pietermaritzburg, South Africa; 8 SAMRC/WITS Developmental Pathways for Health Research Unit, University of the Witwatersrand, Johannesburg, South Africa; 9 Institute of Tropical Medicine, Antwerp, Belgium; 10 University of Milan, Milan, Italy; University College of London, UNITED KINGDOM OF GREAT BRITAIN AND NORTHERN IRELAND

## Abstract

Community-based active case finding (ACF) for tuberculosis (TB) is recommended in high-burden settings, yet evidence on implementation costs from prospective programmatic data remains limited. We estimated and compared the costs of two community-based ACF screening approaches: Computer-Aided Detection of chest X-rays alone (*CAD4TBv7 approach*) and CAD4TBv7 followed by point-of-care C-reactive protein (CRP) testing within a predefined score window (*CAD4TBv7-CRP approach*), evaluated in a prospective, paired screen-positive trial in South Africa and Lesotho. Nested within the TB TRIAGE+ trial (September 2022-September 2024), we applied a bottom-up ingredients-based approach from a programmatic perspective to estimate economic and financial costs, separately for TB-only and integrated (TB, HIV, and non-communicable disease) screening. Uncertainty was estimated via probabilistic sensitivity analysis, while scenario analyses explored higher modelled TB prevalence (1%) and expanded screening volume (100,000 participants). Among 20,023 participants screened (6,986 in South Africa; 13,037 in Lesotho), 72 TB cases were detected under the *CAD4TBv7 approach* and 60 under the *CAD4TBv7-CRP approach* (full analysis set). For TB-only screening, the average cost per TB case detected was USD 5,454 (95% uncertainty interval [UI]: 4,294–6,777) for the *CAD4TBv7 approach* and USD 7,486 (95% UI: 5,948–9,246) for the *CAD4TBv7-CRP approach*, 37% more expensive. Recurrent costs accounted for 67–72% of total costs. Personnel was the dominant cost driver in South Africa, and vehicle costs in Lesotho. Higher TB prevalence would substantially reduce the average cost per TB case detected, whereas scaling up participant numbers at the observed prevalence would not. The additional cost of CRP testing was not offset by savings from averted confirmatory tests, making the *CAD4TBv7-CRP approach* more expensive than *CAD4TBv7* alone. CRP-based sequential screening may offer greater cost advantages in settings with lower inflammatory comorbidity burden, though this requires prospective evaluation. TB screening strategies should be tailored to local epidemiological and health-system contexts.

## Introduction

Tuberculosis (TB) remains a leading cause of infectious disease mortality globally, with an estimated 10·7 million incident cases and 1·23 million deaths in 2024 (WHO, 2025). Approximately one in four people with active TB remains undiagnosed or unreported, leading to increased transmission and delayed treatment initiation [1], [2]. The economic consequences of TB are substantial: TB-related welfare losses in southern Africa are estimated at nearly USD 100 billion, equivalent to 11·6% of regional GDP, with Lesotho among the most severely affected globally [3]. National TB programmes rely predominantly on passive case finding, which may miss individuals who face geographic, socioeconomic, or other barriers to accessing care [4]. Active case finding (ACF) is therefore recommended by WHO in general populations with a TB prevalence of at least 0·5%, and in subpopulations at elevated risk [5]. ACF strategies have also been proven to increase TB detection and reduce community prevalence [6].

Computer-Aided Detection (CAD) of digital chest X-rays is recommended by the WHO as an alternative to human interpretation for TB screening [5]. Point-of-care C-reactive protein (POC-CRP) testing is further recommended as an additional screening tool in people living with human immunodeficiency virus (HIV) in high-burden settings. CAD4TBv7 (Delft Imaging Systems, NL) and LumiraDx POC-CRP (LumiraDx Limited, UK) are commercially available tools implementing these respective approaches. Community-based ACF campaigns place substantial financial demands on health systems, with costs varying considerably by setting, screening algorithm, and programme scale [7], and cost efficiency is highly sensitive to the choice of screening algorithm and underlying TB prevalence. The central hypothesis of the TB TRIAGE+ trial was that, in a sequential algorithm, POC-CRP testing among participants with intermediate CAD scores would selectively reduce the number of expensive confirmatory Xpert tests required, thereby lowering overall screening costs. To our knowledge no study has empirically compared the costs of CAD alone with CAD combined with POC-CRP in a community-based ACF setting [6], [8].

The TB TRIAGE+ trial compared two community-based ACF screening approaches in 20,023 adults across South Africa and Lesotho using a paired screen-positive design: CAD4TBv7 alone (*CAD4TBv7 approach)* and CAD4TBv7 followed by POC-CRP within a predefined score window (*CAD4TBv7-CRP approach*), each with Xpert MTB/RIF Ultra as confirmatory testing. The trial reported, as a co-primary outcome, that the *CAD4TBv7-CRP approach* detected fewer TB cases and was more expensive per TB case detected than the *CAD4TBv7 approach* [9].

In this study, we present a detailed comparative cost analysis of these two screening approaches from a programmatic perspective. We estimate total, capital, and recurrent costs disaggregated by country and cost category, separately for TB-only and integrated (TB, HIV, and non-communicable disease [NCD]) screening, identify the key cost drivers in each setting, and examine how costs vary under alternative scenarios of TB prevalence and screening volume.

## Materials and Methods

### Ethics Statement

The TB TRIAGE+ trial was approved by the National Health Research Ethics Committee in Lesotho (NH-REC, ID52–2022), the Human Sciences Research Council Research Ethics Committee (HSRC REC, REC 2/23/09/20), and the Provincial Health Research Committee of the Department of Health of KwaZulu-Natal (KZ_202209_022) in South Africa. The Ethikkommission Nordwest- und Zentralschweiz in Switzerland confirmed that ethical requirements were met (EKNZ, AO_2022–00044). The trial is registered with the South African National Clinical Trials Register (SANCTR; DOH-27-092022-8096) and ClinicalTrials.gov (NCT05526885). Written informed consent, or witnessed oral consent in the case of illiteracy, was obtained from all participants prior to any trial procedure.

### Study design and setting

The TB TRIAGE+ trial was a prospective, community-based, pragmatic diagnostic study applying a paired screen-positive design, enabling within-person comparison. As per the paired screen-positive design, both screening approaches were applied to the same participants and thus share an identical denominator, differing only in the screening algorithm. Full details of the trial design, setting, and procedures are reported elsewhere [9], [10]. Briefly, the trial was conducted between September 2022 and September 2024 in the Greater Edendale area of Msunduzi Municipality, KwaZulu-Natal, South Africa, and the Butha-Buthe District, Lesotho. Household members aged 18 years or older were eligible; individuals were excluded if seriously ill, currently on TB treatment, or self-reported as pregnant (in Lesotho only, per a country-specific ethics committee requirement).

All participants received a digital chest X-ray analysed by CAD4TBv7. Under the *CAD4TBv7 approach,* participants with scores at or below a predefined threshold were considered TB screen-negative, while those above received confirmatory Xpert Ultra testing. Under the *CAD4TBv7-CRP approach*, participants with scores at or below the lower threshold (≤5 in South Africa, ≤ 10 in Lesotho) were considered TB screen-negative; those with cores within a predefined triage window received additional POC-CRP testing, with Xpert Ultra performed if CRP exceeded 5 mg/L. Those above the upper threshold received Xpert Ultra directly. Site-specific CAD4TBv7 thresholds were pre-specified based on target sensitivity levels but required re-determination during the initial trial phase, as CAD score distributions were found to be highly dependent on the radiological equipment used at each site. Full details are reported in the primary trial paper [9], and in Table A in S1 Appendix. Both approaches were embedded within a broader health campaign including HIV testing and NCD screening for hypertension or diabetes mellitus.

Implementation differed between countries in ways that directly shaped the cost structure. In South Africa, screening was conducted from mobile units that served as both transport and screening sites, remaining stationed at each location for extended periods to cover larger, more densely populated areas. Vehicles were purchased as capital assets and teams comprised senior nursing staff conducting door-to-door household visits within the catchment area of each site. In Lesotho, the mountainous terrain and smaller, more dispersed village sizes necessitated more frequent relocation of screening units. Vehicles were rented weekly and included costs for fuel, insurance, and drivers, with temporary screening sites established using field equipment at each village. Further details are provided in S1 Appendix.

### Data collection

Cost data were collected prospectively throughout implementation and retrospectively from project financial records covering September 2022 to September 2024, which was also the time horizon for the analysis. This analysis adopts a programmatic perspective, considering only costs related to implementation and excluding research-specific expenditures. Both financial and economic costs were estimated.

Resource quantities were measured through field observations, screening logs, and interviews with project managers and screening teams. Unit cost data were obtained from procurement invoices, supplier quotations, and local public healthcare sector salary scales. Costs were recorded in South African Rand (ZAR) or Lesotho Loti (LSL) and converted to 2023 USD using mid-year market exchange rates, appropriate for a programmatic perspective reflecting actual implementer costs (1 ZAR = 1 LSL = 0·05424 USD). Capital costs were annualised and discounted at 3% per WHO-CHOICE guidelines [11]. A full description of cost categories, items, unit costs, and costing equations is provided in Tables E and F in S1 Appendix.

### Outcomes

The primary outcomes were total cost, average cost per participant screened, and average cost per TB case detected under each screening approach, estimated for TB-only screening. TB cases were defined as participants with a positive Xpert Ultra result (excluding trace results) among those indicated for confirmatory testing by the respective screening approach. The cost analysis was conducted on all participants with valid chest X-ray results, irrespective of completeness of downstream data. This differs from the primary analysis set reported in the main trial paper [9], which applied a different pre-specified definition. The resulting difference in TB case counts (72 vs 69 for the *CAD4TBv7 approach*) is explained in S1 Appendix.

Secondary outcomes included the cost breakdown by input category, the proportion of capital versus recurrent costs, and identification of major cost drivers by approach and country. For integrated screening (TB, HIV, and NCD delivered together), total costs and average cost per participant screened are reported overall, as HIV and NCD screening activities were identical across both TB screening approaches and did not vary by algorithm. The marginal cost of adding HIV and NCD screening to TB-only screening was estimated by dividing the difference in total costs between integrated and TB-only screening by the number of additional HIV or NCD positive cases identified, yielding an average marginal cost per additional HIV or NCD case detected.

### Analysis

Total cost was calculated by summing capital and recurrent costs for each screening approach, with average costs per participant screened and per TB case detected derived accordingly. To characterise parameter uncertainty, we performed a probabilistic sensitivity analysis (PSA) with 10,000 Monte Carlo simulations. Full methodological details are provided in S1 Appendix.

We conducted two scenario analyses: the first modelled a higher TB prevalence of 1% [12], while keeping total participants and costs constant; the second scaled screening to 100,000 participants at the observed prevalence, with country-specific numbers reflecting the trial distribution. All analyses were conducted in R version 4·4·1.

The cost analysis was conducted on the full analysis set (all enrolled participants with valid X-ray results), which differs from the primary outcome set reported in the main trial paper.

## Results

### Screening summary

A total of 20,023 participants were screened across South Africa (n = 6,986) and Lesotho (n = 13,037). Under the *CAD4TBv7 approach*, 72 TB cases were detected (31 in South Africa, 41 in Lesotho); under the *CAD4TBv7-CRP approach*, 60 TB cases were detected (29 in South Africa, 31 in Lesotho). The higher screening volume in Lesotho reflected the larger number of eligible villages enumerated, but TB yield was greater in South Africa relative to participants screened.

### Costs

Total economic costs for TB-only screening were USD 392,695 (95% UI: USD 309,144–487,934) for the *CAD4TBv7 approach* and USD 449,175 (95% UI: USD 356,894–554,738) for the *CAD4TBv7-CRP approach* (Table 1). The average cost per participant screened was USD 20 (95% UI: USD 15–24) and USD 22 (95% UI: USD 18–28) respectively. The average cost per TB case detected was USD 5,454 (95% UI: USD 4,294–6,777) for the *CAD4TBv7 approach* and USD 7,486 (95% UI: USD 5,948–9,246) for the *CAD4TBv7-CRP approach,* making the latter 37.3% more expensive. Costs were consistently higher in Lesotho than in South Africa for both approaches: average cost per TB case detected was USD 5,987 (95% UI 4,740–7,411) versus USD 4,749 (95% UI 3,704–5,939) for the CAD4TBv7 approach, and USD 8,544 (95% UI 6,842–10,444) versus USD 6,356 (95% UI 4,993–7,964) for the CAD4TBv7-CRP approach.

**Table 1:** Total costs, cost per screening, and cost per TB case detected by screening approach and country (TB-only screening).

Outcome	CAD4TBv7 approach	CAD4TBv7-CRP approach
**Overall (n = 20,023) | Observed prevalence**		
TB cases detected	72	60
Total cost of TB screening, USD (95% UI)	392,695 [309,144-487,934]	449,175 [356,894-554,738]
Average cost per participant screened, USD (95% UI)	20 [15–24]	22 [18–28]
Average cost per TB case detected, USD (95% UI)	5,454 [4,294-6,777]	7,486 [5,948-9,246]
**South Africa (n = 6,986) | Observed prevalence**		
TB cases detected	31	29
Total cost of TB screening, USD (95% UI)	147,214 [114,818-184,094]	184,325 [144,798-230,966]
Average cost per participant screened, USD (95% UI)	21 [16–26]	26 [21–33]
Average cost per TB case detected, USD (95% UI)	4,749 [3,704-5,939]	6,356 [4,993-7,964]
**Lesotho (n = 13,037) | Observed prevalence**		
TB cases detected	41	31
Total cost of TB screening, USD (95% UI)	245,481 [194,326-303,840]	264,850 [212,096-323,772]
Average cost per participant screened, USD (95% UI)	19 [15–23]	20 [16–25]
Average cost per TB case detected, USD (95% UI)	5,987 [4,740-7,411]	8,544 [6,842-10,444]

Note: Economic costs in 2023 USD. Mean costs are presented with 95% uncertainty intervals (UI) from 10,000 Monte Carlo simulations.

Financial cost estimates are provided in Table K in S1 Appendix. Financial costs were broadly consistent with economic costs: the average financial cost per TB case detected was USD 5,397 for the *CAD4TBv7 approach* and USD 7,243 for the *CAD4TBv7-CRP approach* overall, representing a difference of 34·2%. The difference from economic estimates reflects the exclusion of opportunity costs and inclusion of VAT under financial costing.

### Cost structure and drivers

Recurrent costs accounted for the majority of total costs across both approaches and countries (67·3-72·2%), with a slightly higher recurrent share in Lesotho than South Africa (70·6% vs 67·3%). The unit cost of a single CAD4TBv7 screen was USD 0·80, compared with USD 3·51 for POC-CRP and USD 10·51 for Xpert Ultra. Under the *CAD4TBv7-CRP approach*, the cost of additional CRP testing was not offset by savings from averted Xpert Ultra tests, resulting in higher absolute screening costs than under the *CAD4TBv7 approach*.

**Fig 1:**
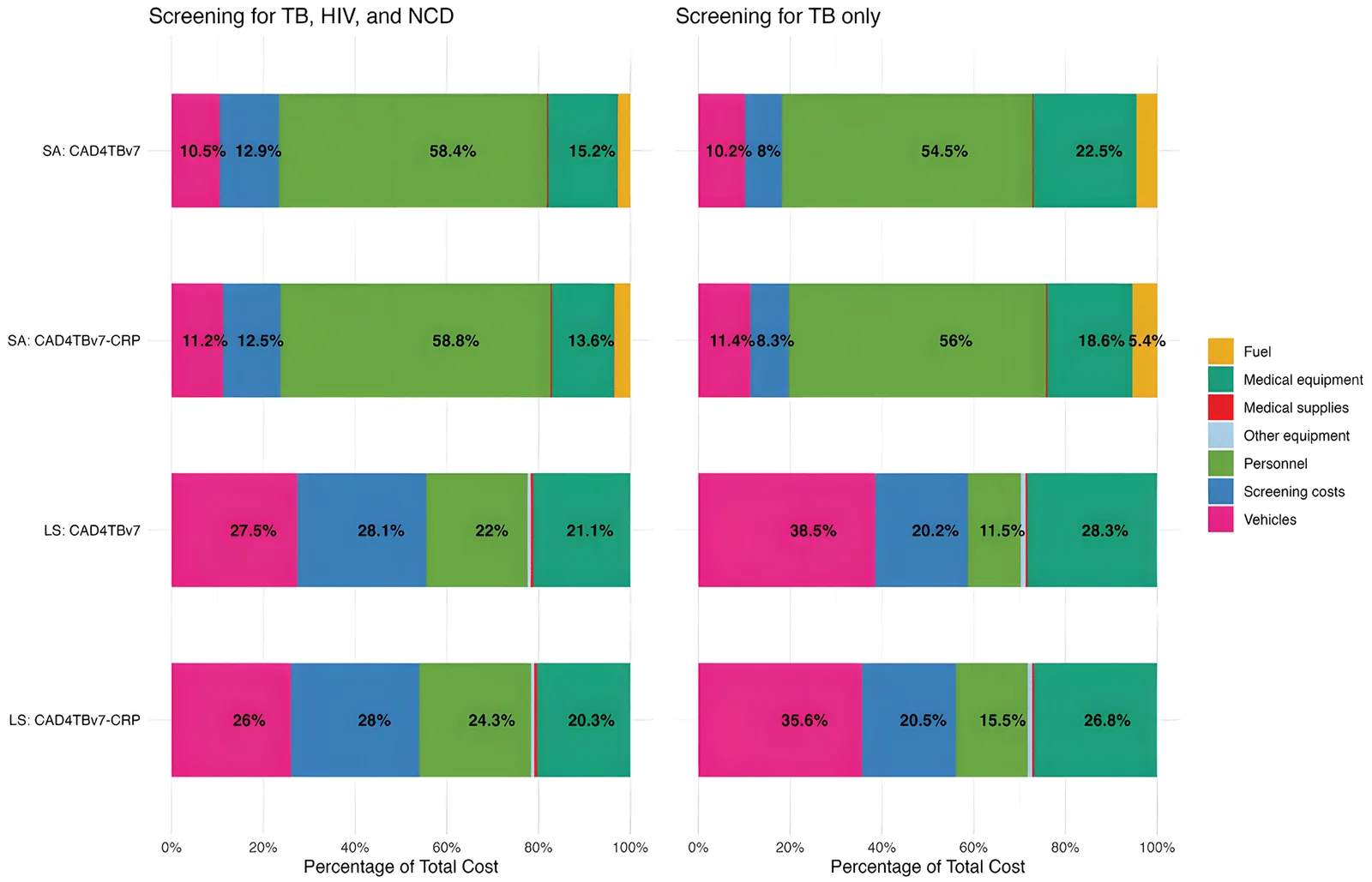
Composition of total costs by approach, country, and screening strategy. Note: Vehicle costs in Lesotho reflect weekly hire charges inclusive of fuel, insurance, and driver expenses. In South Africa, vehicles were purchased as capital assets and fuel is reported as a separate cost category. Direct comparison of vehicle cost components between countries should therefore be interpreted with caution.

Cost drivers differed by country. In South Africa, personnel constituted the largest cost component (54·5-58·8% of total costs), followed by medical equipment. In Lesotho, vehicle rental was the dominant cost driver for TB-only screening (35·6-38·5%). A detailed breakdown of costs by category is shown in Fig 1 and S1 Appendix.

### Integrated screening

When HIV and NCD screening were delivered alongside TB screening, total costs increased to USD 598,355 (95% UI 470,731–743,389) for the CAD4TBv7 approach and USD 654,145 (95% UI 515,375–809,349) for the CAD4TBv7-CRP approach, with 61 HIV cases diagnosed and 3,404 participants screening positive for an NCD across both approaches. Since HIV and NCD screening activities were identical under both approaches, the marginal cost of extending TB-only to integrated screening was estimated across both approaches combined: USD 205,660 spread across 3,465 additional HIV or NCD positive screens, yielding an average of approximately USD 59 per additional HIV or NCD case detected. When accounting for all positive screens across TB, HIV, and NCD combined, the average cost per positive screen was USD 169 (95% UI 133–210) for the CAD4TBv7 approach. This reflects the distribution of shared screening costs across TB, HIV, and NCD cases detected with the same delivery platform, and illustrates the efficiency gains achievable through integrated service delivery. Full country-specific breakdowns are provided in S1 Appendix.

### Scenario analysis

Under the first scenario, modelling a higher TB prevalence of 1% while keeping total participants and costs constant, the cost per participant screened remained unchanged from the primary analysis. However, the average cost per TB case detected decreased substantially to USD 2,887 (95% UI: USD 2,272–3,587) for the *CAD4TBv7 approach* and USD 3,975 (95% UI: USD 3,157-4,907) for the *CAD4TBv7-CRP approach* (Table 2) when compared with the primary analysis. This scenario was motivated by the hypothesis that higher TB prevalence might reduce the cost disadvantage of the *CAD4TBv7-CRP approach*, however it remained dominated across all scenarios.

**Table 2:** Scenario analysis: total costs, cost per screening, and cost per TB case detected by screening approach and country (TB-only screening).

Outcome	CAD4TBv7 approach	CAD4TBv7-CRP approach
**Scenario 1 (Overall, n = 20,023) | 1% modelled TB prevalence**		
TB cases detected	136	113
Total cost of TB screening, USD (95% UI)	392,695 [309,144-487,934]	449,175 [356,894-554,738]
Average cost per participant screened, USD (95% UI)	20 [15–24]	22 [18–28]
Average cost per TB case detected, USD (95% UI)	2,887 [2,272-3,587]	3,975 [3,157-4,907]
**Scenario 2 (Overall, n = 100,000) | Observed prevalence**		
TB cases detected	360	300
Total cost of TB screening, USD (95% UI)	2,098,579 [1,586,876-2,696,285]	2,213,737 [1,689,642-2,815,335]
Average cost per participant screened, USD (95% UI)	21 [16–27]	22 [17–28]
Average cost per TB case detected, USD (95% UI)	5,829 [4,408-7,490]	7,379 [5,632-9,384]
**Scenario 2 (South Africa, n = 34,890) | Observed prevalence**		
TB cases detected	155	145
Total cost of TB screening, USD (95% UI)	1,073,795 [792,124-1,408,336]	1,134,216 [841,483-1,476,115]
Average cost per participant screened, USD (95% UI)	31 [23-40]	33 [24-42]
Average cost per TB case detected, USD (95% UI)	6,928 [5,110-9,086]	7,822 [5,803-10,180]
**Scenario 2 (Lesotho, n = 65,110) | Observed prevalence**		
TB cases detected	205	155
Total cost of TB screening, USD (95% UI)	1,024,784 [794,752-1,287,949]	1,079,521 [848,159-1,339,220]
Average cost per participant screened, USD (95% UI)	16 12 [–]20]	17 [13–21]
Average cost per TB case detected, USD (95% UI)	4,999 [3,877-6,283]	6,965 [5,472-8,640]

*Note: These are economic costs represented in 2023 USD. Mean of the sampled costs are presented along with their 95% uncertainty intervals (UI). Scenario 1: TB prevalence modelled at 1% with participant numbers and total costs held constant at observed values. Country-level results for scenario 1 are not presented separately. Since total costs and participant numbers are held constant, country-specific estimates reduce proportionally from the primary analysis in line with the modelled increase in TB yield. Scenario 2: screening volume scaled to 100,000 participants at observed prevalence, with country proportions reflecting the trial distribution.*

Under the second scenario, scaling screening to 100,000 participants at the observed prevalence, the average cost per participant screened remained similar to the primary analysis (USD 21–29 depending on the approach and strategy), and the average cost per TB case detected was likewise comparable. Costs were higher in Lesotho than South Africa under both scenarios, consistent with the primary analysis. Across all scenarios, the *CAD4TBv7-CRP approach* remained dominated by the *CAD4TBv7 approach*.

Across both countries and screening strategies, empirical cost estimates fell within the 95% uncertainty intervals of the PSA-sampled means, confirming consistency between the deterministic and probabilistic estimates (S1 Appendix).

## Discussion

In this prospective, two-country cost analysis nested within the TB TRIAGE+ trial, the CAD4TBv7 approach was less costly and detected more TB cases than the CAD4TBv7-CRP approach, with the latter 37% more expensive per TB case detected. Recurrent costs dominated expenditure across both approaches and countries, accounting for 67–72% of total costs. Personnel was the primary cost driver in South Africa, and vehicle hire dominated in Lesotho. Scenario analyses showed that the average cost per TB case detected was highly sensitive to underlying TB prevalence but not to screening volume. This reflects the dominance of recurrent costs, which scale with the number of participants screened rather than with the number of cases detected.

The dominance of personnel costs in South Africa is consistent with prior evidence from combined TB-HIV ACF in the same setting, where personnel represented the largest cost component [13]. The high vehicle costs in Lesotho reflect the weekly hire model including fuel, insurance, driver costs. Capital costs in Lesotho were also higher due to equipment being assigned 100% to the campaign, compared with 80% in South Africa where equipment was shared with other services. Longer specimen transport distances to the central laboratory further raised the logistical costs in Lesotho. The high recurrent cost share observed across both settings is consistent with the broader ACF costing literature, which identifies recurrent costs as the dominant expenditure category, with the relative contribution varying by screening volume [14]– [15].

We estimated an average cost per person screened of USD 20–22 for TB-only screening. This is consistent with estimates from comparable ACF campaigns, including USD 21 in Zambia [16] using Xpert Ultra and USD 21–22 for CRP or chest X-ray (CXR)-based screening in Uganda [8]. Our estimated average cost per TB case detected is broadly comparable to estimates from similar community-based ACF campaigns in South Africa, where a CAD-directed Xpert Ultra screening strategy has been estimated at USD 2,207-3,745 per TB case detected depending on the threshold applied [12], and to USD 8,400 per TB case detected for universal Xpert Ultra in Uganda [17]. The average cost per TB case detected was highly sensitive to the underlying TB prevalence, which is consistent with evidence from a comparable ACF campaign [17]. In contrast, scaling screening volume to 100,000 participants at the observed prevalence did not significantly reduce average costs, reflecting the limited scope for economies of scale when recurrent costs dominate total expenditure. However, this pattern differed by country. Average costs per participant screened increased in South Africa but decreased in Lesotho under scaled screening. This likely reflects the different dominant cost drivers in each setting, as personnel costs in South Africa scale proportionally with participants screened, whereas vehicle hire costs in Lesotho, which are partly fixed per village visit, are spread across a larger number of participants under scaled screening, yielding modest efficiency gains.

The higher costs of the CAD4TBv7-CRP approach relative to the CAD4TBv7 approach may be explained by the failure of CRP screening to sufficiently avert downstream Xpert Ultra testing to offset its own cost. CRP has demonstrated strong performance as a TB screening tool among care-seeking people living with HIV (PLHIV) and has been recommended by WHO for TB screening in PLHIV attending outpatient clinics [18,19]. However, the TB TRIAGE+ trial screened a general community population rather than exclusively PLHIV. Additionally, in the trial’s pre-specified analysis, TB case yield showed no effect modification by HIV status: Among PLHIV, the *CAD4TBv7-CRP approach* detected fewer TB cases than *CAD4TBv7* alone (16 vs 18 of 20 cases), and TB prevalence among PLHIV (0.40%) was close to that of the overall population [9]. Restricting CRP-based screening to PLHIV would therefore not have improved case detection in this community setting.

The high prevalence of conditions associated with systemic inflammation in our study settings (including HIV infection, elevated haemoglobin A1c and blood pressure) may have reduced CRP specificity for TB and thereby limited its ability to avert confirmatory testing [20–22]. However, this remains a hypothesis requiring prospective evaluation. CRP-based sequential screening may offer greater cost advantages in settings with a lower comorbidity burden, though this warrants further investigation [23].

The integrated screening results highlight an important efficiency argument for bundled service delivery. Although total costs increased when HIV and NCD screening were added to TB screening, the average cost per positive screen across TB, HIV, and NCD combined was USD 169 for the *CAD4TBv7 approach*, and the marginal cost of detecting an additional HIV or NCD case was approximately USD 59, compared with USD 137 per diagnosis reported for standalone community health worker-led NCD screening in the same Lesotho district under routine implementation conditions [24]. This contrast illustrates the efficiency gains achievable by integrating additional health services within an existing screening infrastructure rather than running parallel vertical programmes and supports the programmatic case for bundling TB screening with NCD and HIV services in high-burden settings. Efficiency gains might be strongest in settings with low community TB prevalence. In our study setting, vertical TB-only screening was costly per case detected and did not become more efficient with expanded screening volume due to the high share of recurrent costs, so bundling HIV and NCD services onto existing screening infrastructure yields greater returns than expanding TB-only volume.

This study has several strengths. It presents prospective, empirical cost data from the same intervention implemented simultaneously in two distinct health-system contexts, enhancing the external relevance of the findings and providing direct insight into how delivery models shape cost structures. Costs were estimated using a bottom-up ingredients-based approach with resource quantities measured prospectively through field observations, screening logs, and staff interviews, and unit costs sourced from procurement invoices and local salary scales. This approach significantly improves reproducibility and transferability to other settings. The pre-specified probabilistic sensitivity analysis quantifies parameter uncertainty and confirms that the cost advantage of the *CAD4TBv7 approach* over *CAD4TBv7-CRP approach* is maintained across the full range of simulated inputs. This finding is also insensitive to the TB case definition. Counting Xpert trace results as TB cases leaves the ordering of the two approaches by cost per TB case detected unchanged (9). Under the paired screen-positive design, any trace case detected under the *CAD4TBv7-CRP approach* is also detected under the *CAD4TBv7 approach*, such that the former remains dominated regardless of the case definition applied. Finally, reporting costs separately for TB-only and integrated screening provides evidence relevant both to programmes focusing exclusively on TB and to those delivering bundled services.

A limitation is that cost estimates were obtained in a trial context and may not fully reflect routine programmatic implementation, where costs may differ due to variation in staffing arrangements, supply chains, infrastructure, and operational efficiency. Generalisation to settings with substantially different delivery models, staffing structures, and TB prevalence should thus be done with caution.

Community-based TB screening strategies should be tailored to local epidemiological and health-system contexts, considering TB prevalence, comorbidity burden, delivery model, and the scope of integrated services for estimating costs and selecting screening algorithms. In high-comorbidity settings such as South Africa and Lesotho, CAD-only screening offered a more cost-efficient approach than CAD combined with POC-CRP. The high recurrent cost share observed in both settings limits the potential for economies of scale but underscores the efficiency gains achievable through integration with existing health campaigns. Future research should link cost estimates to health outcomes within a decision-analytic framework, which would also clarify whether vertical TB-only ACF is warranted in low prevalence settings, and examine cost-minimising threshold combinations for CAD and CRP in ACF settings.

## Supporting information

S1 Appendix Table A in S1 Appendix. Site-specific CAD4TBv7 score thresholds. Table B in S1 Appendix. Medical equipment items. Table C in S1 Appendix. Medical supplies. Table D in S1 Appendix. Economic versus financial costs. Table E in S1 Appendix. Purchase cost summary. Table F in S1 Appendix. Cost inputs. Table G in S1 Appendix. Average value of capital and recurrent costs and their share of total costs by country and screening intervention (n = 20,023). Table H in S1 Appendix. Total costs and cost per TB case detected by screening approach and country: integrated screening (TB + HIV + NCD), primary analysis. Table I in S1 Appendix. Scenario analysis: total costs and cost per TB case detected by screening approach and country: integrated screening (TB + HIV + NCD). Table J in S1 Appendix. Total and mean cost per TB, NCD, or HIV case observed by country and intervention arm. Table K in S1 Appendix. Financial cost outcomes by approach, delivery, and country. Table L in S1 Appendix. Total costs and cost per screening and TB case detected when administering Xpert Ultra for all under different scenarios (post-hoc). Table M in S1 Appendix. Cost per TB case detected under alternative case denominators. Fig A in S1 Appendix. Drivers of overall costs by approach and country for screening TB. Fig B in S1 Appendix. Drivers of overall costs by approach and country for screening TB, HIV, and NCD. Fig C in S1 Appendix. Probabilistic sensitivity analysis of total screening costs by intervention arm and country.
(DOCX)

## References

[1] Lönnroth K, Castro KG, Chakaya JM, Chauhan LS, Floyd K, Glaziou P. Tuberculosis control and elimination 2010–50: cure, care, and social development. The Lancet. 2010;375(9728):1814–29. doi: 10.1016/S0140-6736(10)60483-7 20488524

[2] World Health Organization. Global tuberculosis report. Geneva: World Health Organization. 2025. https://iris.who.int/server/api/core/bitstreams/e97dd6f4-b567-4396-8680-717bac6869a9/content

[3] Wang J-C, Zuo Q-Y, Zheng J-X, Lv W-W, Wang W, Zhang S-X. The Global Economic Burden of Tuberculosis: Regional Disparities and Implications. Open Forum Infect Dis. 2026;13(1):ofag014. doi: 10.1093/ofid/ofag014 41626303 PMC12852480

[4] Teibo TKA, Andrade RL de P, Rosa RJ, de Abreu PD, Olayemi OA, Alves YM, et al. Barriers That Interfere with Access to Tuberculosis Diagnosis and Treatment across Countries Globally: A Systematic Review. ACS Infect Dis. 2024;10(8):2600–14. doi: 10.1021/acsinfecdis.4c00466 39023509 PMC11320565

[5] World Health Organization. WHO Consolidated Guidelines on Tuberculosis. Module 2: Screening - Systematic Screening for Tuberculosis Disease. 1st ed. Geneva: World Health Organization; 2021. 1 p. 33822560

[6] Burke RM, Nliwasa M, Feasey HRA, Chaisson LH, Golub JE, Naufal F, et al. Community-based active case-finding interventions for tuberculosis: a systematic review. Lancet Public Health. 2021;6(5):e283–99. doi: 10.1016/S2468-2667(21)00033-5 33765456 PMC8082281

[7] Laurence YV, Griffiths UK, Vassall A. Costs to health services and the patient of treating tuberculosis: a systematic literature Review. Pharmacoeconomics. 2015;33(9):939–55. doi: 10.1007/s40273-015-0279-6 25939501 PMC4559093

[8] Murray M, Cattamanchi A, Denkinger C, Van’t Hoog A, Pai M, Dowdy D. Cost-effectiveness of triage testing for facility-based systematic screening of tuberculosis among Ugandan adults. BMJ Glob Health. 2016;1(2):e000064. doi: 10.1136/bmjgh-2016-000064 28588939 PMC5321327

[9] Reither K, Vanobberghen F, Verjans A, Harkare HV, Jacobs BKM, Heerden A van, et al. Community-based tuberculosis screening with computer-aided detection technology alone and in combination with point-of-care C-reactive protein testing: a paired screen-positive trial. Lancet Infect Dis. 2026;S1473-3099(26)00222-7. doi: 10.1016/S1473-3099(26)00222-7 42385764

[10] Signorell A, van Heerden A, Ayakaka I, Jacobs BK, Antillon M, Tediosi F, et al. Effectiveness and cost-effectiveness of community-based TB screening algorithms using computer-aided detection (CAD) technology alone compared with CAD combined with point-of-care C reactive protein testing in Lesotho and South Africa: protocol for a paired screen-positive trial. BMJ Open. 2025;15(7):e093989. doi: 10.1136/bmjopen-2024-093989 40721262 PMC12306269

[11] World Health Organization. WHO-CHOICE. https://www.who.int/news-room/questions-and-answers/item/who-choice-frequently-asked-questions. 2014. Accessed 2023 October 1.

[12] Scott AJ, Perumal T, Pooran A, Oelofse S, Jaumdally S, Swanepoel J, et al. Clinical evaluation of computer-aided digital x-ray detection of pulmonary tuberculosis during community-based screening or active case-finding: a case-control study. Lancet Glob Health. 2025;13(3):e517–27. doi: 10.1016/S2214-109X(24)00516-3 40021309

[13] Kranzer K, Lawn SD, Meyer-Rath G, Vassall A, Raditlhalo E, Govindasamy D. Feasibility, yield, and cost of active tuberculosis case finding linked to a mobile HIV service in Cape Town, South Africa: a cross-sectional study. PLOS Med. 2012;9(8):e1001281. doi: 10.1371/journal.pmed.1001281 PMC3413719 22879816

[14] Alsdurf H, Empringham B, Miller C, Zwerling A. Tuberculosis screening costs and cost-effectiveness in high-risk groups: a systematic review. BMC Infect Dis. 2021;21(1):935. doi: 10.1186/s12879-021-06633-3 34496804 PMC8425319

[15] Jo Y, Mirzoeva F, Chry M, Qin ZZ, Codlin A, Bobokhojaev O, et al. Standardized framework for evaluating costs of active case-finding programs: An analysis of two programs in Cambodia and Tajikistan. PLoS One. 2020;15(1):e0228216. doi: 10.1371/journal.pone.0228216 31986183 PMC6984737

[16] Jo Y, Kagujje M, Johnson K, Dowdy D, Hangoma P, Chiliukutu L, et al. Costs and cost-effectiveness of a comprehensive tuberculosis case finding strategy in Zambia. PLoS One. 2021;16(9):e0256531. doi: 10.1371/journal.pone.0256531 34499668 PMC8428570

[17] Baik Y, Nakasolya O, Isooba D, Mukiibi J, Kitonsa PJ, Erisa KC, et al. Cost to perform door-to-door universal sputum screening for TB in a high-burden community. Int J Tuberc Lung Dis. 2023;27(3):195–201. doi: 10.5588/ijtld.22.0567 PMC10193835 36855034

[18] Yoon C, Semitala FC, Atuhumuza E, Katende J, Mwebe S, Asege L, et al. Point-of-care C-reactive protein-based tuberculosis screening for people living with HIV: a diagnostic accuracy study. Lancet Infect Dis. 2017;17(12):1285–92. doi: 10.1016/S1473-3099(17)30488-7 28847636 PMC5705273

[19] Dhana A, Gupta RK, Hamada Y, Kengne AP, Kerkhoff AD, Yoon C, et al. Clinical utility of WHO-recommended screening tools and development and validation of novel clinical prediction models for pulmonary tuberculosis screening among outpatients living with HIV: an individual participant data meta-analysis. Eur Respir Rev. 2023;32(168). doi: 10.1183/16000617.0021-2023 PMC10245131 37286216

[20] Fernández LG, Firima E, Gupta R, Sematle MP, Khomolishoele M, Molulela M, et al. Awareness, treatment, and control among adults living with arterial hypertension or diabetes mellitus in two rural districts in Lesotho. PLOS Glob Public Health. 2024;4(9):e0003721. doi: 10.1371/journal.pgph.0003721 39348361 PMC11441678

[21] Singh U, Olivier S, Cuadros D, Castle A, Moosa Y, Zulu T, et al. The met and unmet health needs for HIV, hypertension, and diabetes in rural KwaZulu-Natal, South Africa: analysis of a cross-sectional multimorbidity survey. Lancet Glob Health. 2023;11(9):e1372–82. doi: 10.1016/S2214-109X(23)00239-5 37591585 PMC10447220

[22] Samuels THA, Wyss R, Ongarello S, Moore DAJ, Schumacher SG, Denkinger CM. Evaluation of the diagnostic performance of laboratory-based c-reactive protein as a triage test for active pulmonary tuberculosis. PLoS One. 2021;16(7):e0254002. doi: 10.1371/journal.pone.0254002 34252128 PMC8274836

[23] Derendinger B, Mochizuki TK, Marcelo D, Shankar D, Mangeni W, Nguyen H, et al. C-Reactive Protein-based Screening of People with Tuberculosis Symptoms: A Diagnostic Accuracy Study. Am J Respir Crit Care Med. 2025;211(3):499–506. doi: 10.1164/rccm.202405-1000OC 39642368 PMC11936131

[24] Spaolonzi N, Gupta R, Sanchez-Samaniego G, Chammartin F, Carter AW, Tahirsylaj T, et al. Costs and coverage of community health worker-led hypertension and diabetes screening in rural Lesotho (ComBaCaL): cost–consequence analysis of a cohort study. The Lancet Primary Care. 2025;1(4):100034. doi: 10.1016/j.lanprc.2025.100034

