# Supplementary material for: Comparative cost analysis of community-based tuberculosis screening: Computer-Aided Detection (CAD) alone versus CAD with point-of-care C-reactive protein testing in South Africa and Lesotho
Source: PLOS Glob Public Health. 2026 Sep 21;6(9):e0007341. doi: 10.1371/journal.pgph.0007341 (PMC13592630; doi:10.1371/journal.pgph.0007341)
Supplement: Supplementary file 1 [file pgph.0007341.s001.docx]

# **S1 Appendix**

Comparative cost analysis of community-based tuberculosis screening: Computer-Aided Detection (CAD) alone versus CAD with point-of-care C-reactive protein testing in South Africa and Lesotho

Harsh Vivek Harkare^*^, Anna Verjans^*^, Phillip Joseph, Mashaete Kamele, Shriya Misra, Mamatlakeng Keitseng, Mojabeng Mabeleng, Thandanani Madonsela, Alastair van Heerden, Johanna Kurscheid, Bart K.M. Jacobs, Aita Signorell, Fiona Vanobberghen, Klaus Reither, Irene Ayakaka, Shannon Bosman, Fabrizio Tediosi

Corresponding author: Harsh Vivek Harkare, [harshvivek.harkare@swisstph.ch](mailto:harshvivek.harkare@swisstph.ch)

Supplementary methods …………………………………………………	2

Trial setting and implementation ………………………………………	2

Cost categories and items ………………………………………………	4

Costing function ………………………………………………………..	9

Unit costs ……………………………………………………………….	10

Scenario analysis ……………………………………………………….	14

Probabilistic sensitivity analysis ……………………………………….	14

Supplementary results ……………………………………………………	15

Capital and recurrent costs ……………………………………………..	15

Integrated screening costs ………………………………………………	15

Cost drivers …………………………………………………………….	19

Financial costs …………………………………………………………	20

Scenario analysis ………………………………………………………	20

Sensitivity analysis …………………………………………………….	21

References ……………………………………………………………….	22

# Methods

## Trial setting and implementation

The TB TRIAGE+ trial was conducted between September 2022 and September 2024 at two sites: the Greater Edendale area of Msunduzi Municipality, KwaZulu-Natal, South Africa, and the Butha-Buthe District, Lesotho. Implementation differed substantially between sites in ways that directly shaped the cost structure of each screening approach. Site-specific CAD4TBv7 score thresholds for each screening approach are shown in Table A.

**Table A. Site-specific CAD4TBv7 score thresholds**

|  | **South Africa** | **Lesotho** |
| --- | --- | --- |
| CAD4TBv7 approach |  |  |
| Screen-negative (no further testing) | ≤13 | ≤25 |
| Referred for Xpert Ultra | >13 | >25 |
| CAD4TBv7-CRP approach |  |  |
| Screen-negative (no further testing) | ≤5 | ≤10 |
| CRP triage window (POC-CRP performed) | >5 to ≤44 | >10 to ≤50 |
| Direct Xpert Ultra (above window) | >44 | >50 |
| CRP threshold for Xpert Ultra referral | >5 mg/L | >5 mg/L |

### **Delivery model**

In South Africa, the implementing partner purchased a fleet of vehicles comprising two IVECO Daily vans, two Ford Rangers, and two three-room caravans. The caravans and IVECO vans functioned as mobile consultation units, providing on-site space for screening procedures and sputum analysis using Xpert Ultra machines. The campaign operated four days per week, with vehicles and equipment assumed to be allocated 80% to the campaign. Screening teams comprised one senior nurse and several technicians. Managerial and administrative staff supported the campaign at an estimated level of effort ranging from 2·5% to 20% of their programmatic time. Fuel and driver costs were recorded separately from vehicle capital costs.

In Lesotho, vehicles were rented on a weekly basis (one Toyota Fortuner and one Mercedes Sprinter Twin cab) with rental costs inclusive of fuel, insurance, and drivers. Unlike South Africa, vehicles in Lesotho were used solely for transporting personnel, equipment, and consumables; they did not serve as screening sites. The mountainous terrain and smaller, more dispersed village sizes necessitated weekly or even more frequent relocation of the screening team, requiring the procurement of additional field equipment including tents, tables, chairs, and heaters to establish temporary screening sites at each location. The campaign operated five days per week, with screening teams comprising junior nursing assistants. Equipment was allocated 100% to the campaign. For a five-week period during which the team operated in a separate district, overnight allowances were included in personnel costs.

### Ethics approval

The trial was approved by the National Health Research Ethics Committee in Lesotho (NH-REC, ID52-2022), the Human Sciences Research Council Research Ethics Committee (HSRC REC, REC 2/23/09/20) and the Provincial Health Research Committee of the Department of Health of KwaZulu-Natal (KZ_202209_022) in South Africa. Additionally, the ethics commission of Northwestern- and Central-Switzerland (EKNZ) provided a statement confirming the trial meets all ethical requirements for a Swiss research project (EKNZ, AO_2022-00044).

### Full analysis set

The primary trial paper (Reither et al., in press) reports TB case yields based on the primary analysis set, which excludes participants with missing outcomes under either screening approach. The cost analysis presented in this manuscript was conducted on the full analysis set (FAS), defined as all enrolled participants with a valid chest X-ray result, irrespective of data completeness for downstream screening steps.

Under the FAS definition, 72 TB cases were detected under the *CAD4TBv7 approach* and 60 under the *CAD4TBv7-CRP approach*. The corresponding figures in the primary analysis set reported in the trial paper are 69 and 60, respectively. The difference of three cases in the *CAD4TBv7 approach* reflects participants who had a valid X-ray result and a positive Xpert Ultra result under that approach but lacked complete data under the *CAD4TBv7-CRP approach* and were therefore excluded from the primary analysis set.

The FAS was used for the cost analysis as it captures all participants for whom screening costs were incurred, providing the most complete and accurate denominator for cost estimation. Only Xpert Ultra results obtained among participants indicated for confirmatory testing by the respective screening algorithm were counted; results obtained outside the algorithm (for example, under a separate national algorithm in Lesotho) were excluded.

## Cost categories and items

### Capital costs

Capital costs include those items that can be used for more than one year i.e. vehicles, medical and other equipment lasting for more than one year, or equipment that is done as a one-off item. To calculate the economic cost of these items we apply a discount rate of 3%, in line with WHO-CHOICE guidelines (WHO, 2014). Financial costs include VAT of 15%. For South Africa (SA) all the capital costs already include delivery costs, while for Lesotho (LS) delivery costs are included where applicable.

#### Vehicles

The economic cost of vehicles includes the purchase price of each vehicle, annualised over its expected lifespan and discounted accordingly. In SA, it is assumed that vehicles were used 80% of the time for the campaign, which operated four days per week. The fleet comprised two IVECO Daily vans, two Ford Rangers, and two three-room caravans. The caravans and IVECO vans served as mobile consultation units, providing space for screening procedures and on-site analysis of sputum samples using Xpert Ultra machines. Operating costs for fuel and drivers in SA were calculated separately.

#### Medical equipment

The cost of medical equipment encompassed various diagnostic devices and machinery, including X-ray machines (SA: FUJIFILM FDR Smart (FujiFilm, Japan) with a FXRD 1717V panel detector; LS: battery-operated portable Delft Light machine (Delft Imaging System, NL) with Canon CXDI-701 wireless flat panel detector), Xpert Ultra instruments, CAD4TB boxes, laptops, LumiraDx machines, and other general diagnostic tools such as blood pressure monitors and thermometers. In LS, an additional Xpert Ultra machine was procured relative to SA to support planned field activities. To derive the annualised cost, the purchase price of each item is adjusted using the discount factor. A detailed list of the equipment and their respective quantities used in each country is provided in Table B.

**Table B. Medical equipment items**

| **South Africa** | | **Lesotho** | |
| --- | --- | --- | --- |
| **Item** | **#** | **Item** | **#** |
| X-ray unit | 1 | X-ray unit | 1 |
| Xpert MTB/RIF Ultra | 1 | Xpert MTB/RIF Ultra | 2 |
| Printer Xpert MTB/RIF Ultra | 1 | Printer Xpert MTB/RIF Ultra | 2 |
| Scanner Xpert MTB/RIF Ultra | 1 |  |  |
| UPS Xpert MTB/RIF Ultra | 1 | UPS Xpert MTB/RIF Ultra | 2 |
| CAD4TB box | 1 | CAD4TB box | 1 |
| Laptop CAD4TB | 1 | Laptop CAD4TB | 1 |
| LumiraDx | 2 | LumiraDx | 2 |
| Lead apron (0.25 Pb) | 4 | Lead apron (0.35 Pb) | 1 |
|  |  | Thyroid shield* | 3 |
|  |  | Battery detector | 2 |
|  |  | Detector cable | 1 |

*Note: *Costs with an asterisk are not available for LS and therefore borrowed from SA. Cost of Xpert MTB/RIF Ultra is inclusive of a laptop and software costs. LumiraDx costs are inclusive of the scanner.*

#### Other equipment (Lesotho)

For LS, an additional category, ‘other equipment,’ is designated to encompass items such as tents, tables, chairs, and heaters. In LS tents were set up and served as consultation rooms where screening tests were performed and sputum samples analysed.

### Recurrent costs

Recurrent costs occur annually and include personnel, screening costs (test kits), medical supplies (consumables), fuel cost, and the hiring cost of vehicles (LS only). For these costs, no discount factor is applied, but financial costs still include VAT. For screening and medical supplies costs the delivery cost is either based on available data, using the freight cost per box, or assumed to be 2%, based on the average delivery cost of known items. In addition, for the screening costs also the number of repeated tests is considered to calculate the cost.

#### Personnel cost

Personnel cost is determined based on the monthly salaries of screening teams, according to local public healthcare sector wages, along with managerial, administrative, and office support staff in SA. Screening teams were assumed to dedicate 100% of their time to the campaign in LS and 80% in SA, reflecting the number of field days. Since administrative and managerial staff are not exclusively assigned to a single project but allocate their time across multiple initiatives, SA provided an estimate of the level of effort (LOE) each individual programmatically devotes to the campaign, ranging from 2.5% to 20%. The same LOE estimates are applied to known managerial roles in LS. Additionally, for LS, allowances are included for a duration of five weeks, when the team conducted the campaign in another district, requiring them to stay overnight for five weeks.

#### Screening cost

Screening costs are dependent on the number of individuals screened in each country. Therefore, this cost is informed by the actual usage of test kits recorded during data collection, accounting for both completed and repeated tests to estimate costs for 2023-24. The analysis includes the following screening items: HbA1c test kits, CRP test kits, Xpert Ultra cartridges, HIV rapid tests (Alere Determine HIV 1/2 test), HIV confirmatory tests (Uni-Gold Recombigen HIV 1/2 test), and CAD4TB imaging. A uniform unit cost is applied for all items across both countries.

#### Medical supplies

The consumables required for screening procedures (such as alcohol swabs) and medical equipment such as lancets were assigned costs based on their usage per participant or per procedure. Most items had the same cost assumptions for both SA and LS, except for cases where specific cost data were available for LS. Notably, X-ray gowns were only used for female participants in SA. Additionally, items such as sputum cups were sourced from hospital supplies in LS, where specific cost data were unavailable. A comprehensive list of all consumables and their cost assumptions is provided in Table C.

**Table C. Medical supplies**

| **Item** | **#** | **Cost assignment** |
| --- | --- | --- |
| **Alcohol swabs** | Assumed to use 1 per procedure per participant (HbA1c, CRP, HIV) | Same cost used for both countries (no package size available for LS) |
| **Sputum cups** | 1 used per person for Xpert | Same cost for both countries (no data available for LS: from the hospital) |
| **Lancets** | 1 used per procedure (HbA1c, CRP and HIV) | Cost available for both countries |
| **Gowns X-ray – only SA** | 1 for each female participant | Cost available for both countries |

#### Fuel cost

Monthly fuel cost per vehicle for SA specific for the screening campaign is provided by the implementing partner. For LS, fuel cost is included in the hiring cost of vehicles.

A summary of the cost categories, with a description of how the economic and financial costs are estimated, is provided in Table D.

**Table D. Economic vs financial costs**

| **Cost component** | **Economic cost** | **Financial cost**  **(includes VAT)** |
| --- | --- | --- |
| **Capital equipment** | | |
| **Vehicles** | SA: 80% of annualised purchasing cost (discounted)  LS: hiring cost | SA: 100% of annualised purchasing cost  (not discounted) |
| **Medical equipment** | SA: 80% of annualised purchasing cost (discounted)  LS: annualised purchasing cost (discounted) | SA: 100% of annualised purchasing cost  LS: annualised purchasing cost |
| **Other equipment** | LS: annualised purchasing cost (discounted) | LS: annualised purchasing cost |
| **Annual recurrent cost** | | |
| **Vehicles** | LS: hiring cost | LS: hiring cost |
| **Personnel** | SA: monthly salary considering estimated programmatic time  LS: monthly salary considering estimated programmatic time + allowance | SA: monthly salary considering estimated programmatic time  LS: monthly salary considering estimated programmatic time + allowance |
| **Medical supplies** | Cost measured by usage per person | Cost measured by number of packages |
| **Screening** | Cost of used test kits/cartridges | Cost of number of boxes of test kits/cartridges needed |
| **Fuel cost** | Annualised fuel cost | Annualised fuel cost |

Financial and economic costs were distinguished as follows. Financial costs represent the actual expenditure incurred by the programme, inclusive of value-added tax (VAT, 15%); capital items were entered at their VAT-inclusive purchase price and depreciated on a straight-line basis over their useful life. Economic costs reflect the opportunity cost of the resources used and differ in three respects. First, VAT was excluded, as it is a transfer payment rather than a use of real resources. Second, capital items (medical and diagnostic equipment and vehicles) were valued at their equivalent annual cost: the VAT-exclusive, CPI-adjusted purchase price was annualised over the item's useful life using a 3% discount rate and weighted by the proportion of the item's capacity allocated to the campaign. This discounted annualisation is the opportunity-cost valuation of capital, capturing the value forgone by committing funds to durable assets over their service life rather than the cash outlay in the year of purchase. Personnel were valued at full salary according to national public-sector pay scales and the level of effort each cadre devoted to the campaign. Thus, economic and financial personnel costs were equal. Recurrent items (diagnostic tests, consumables, and fuel) were valued at the prices paid. All costs were uprated to 2023 using the World Bank consumer price index and converted to USD at 1 ZAR = 1 LSL = 0.05424.

Costs were recorded in South African Rand (ZAR) or Lesotho Loti (LSL) and converted to 2023 USD using mid-year market exchange rates (1 ZAR = 1 LSL = 0·05424 USD; source: OANDA).

## Costing function

The total annual cost, cost per person screened, and cost per person diagnosed with TB for the integrated screening model is calculated as per the following costing function:

$$Total trial cost= Fixed cost for screening over trial period +\left( Variable cost of CAD screening*prop screened*\#screened \right)+\left( Variable cost CRP*prop screened*\#screened \right)+\left( Variable cost HIV rapid screening*prop screened*\#screened \right)+(Variable cost HIV conf screening*prop screened*\#screened)+\left( Variable cost Xpert testing*prop screened*\#screened \right) +\left( Variable cost NCD screening*prop screened*\#screened \right) +(Variable cost gowns*prop women*\#screened)$$

Note: Each of the variable costs are calculated by multiplying the cost per screening test with the proportion (prop) of participants that received the specific test and multiplying this proportion with the total number of people screened per year. The cost for gowns is only applicable for SA.

The fixed cost component of the total trial cost is calculated by doubling the annual fixed cost. Variable costs are determined based on the proportion of the screened population that underwent testing, as specified by trial guidelines for each screening strategy. Consequently, this may not always correspond to the actual number of tests performed, as stock shortages occasionally prevented some tests from being conducted.

Formulae for cost per screening and cost per diagnosis:

$$Cost per screening= \frac{Total trial cost}{Total number of people screened}$$

$$Cost per diagnosis = \frac{Total trial cost}{Total number of people diagnosed as per Xpert Ultra}$$

## Unit costs

**Table E. Purchase cost summary**

| **Equipment** | **Country** | **Model / detail** | **Qty** | **Purchase price (USD, incl. VAT)** |
| --- | --- | --- | --- | --- |
| Chest X-ray | South Africa | FUJIFILM FDR Smart (FXRD 1717V detector) | 1 | $76,019 |
|  | Lesotho | Delft Light (Canon CXDI-701 detector) | 1 | $88,174 |
| CAD4TB box + software | South Africa | CAD4TBv7 | 1 | $6,651 |
|  | Lesotho | CAD4TBv7 | 1 | $6,986 |
| LumiraDx platform | South Africa | Instrument × 2 | 2 | $8,778 |
|  | Lesotho | Instrument × 2 (+2 barcode scanners $877) | 2 | $7,700 |
| Xpert MTB/RIF Ultra | South Africa | incl. laptop + software × 1 | 1 | $24,412 |
|  | Lesotho | incl. laptop + software × 2 | 2 | $49,676 |

*Note: USD conversion at 1 ZAR = 1 LSL = 0.05424 USD (2023). Lesotho X-ray/CAD prices originate in EUR (Delft) and were converted via LSL in the costing tool.*

Prices are the one-time purchase cost including VAT, expressed in 2023 USD. For items without a source document, no invoice was available on record, but numbers were present in base costing tool.

**Table F. Cost inputs**

| **Cost category** | **Item** | **Annualised cost (2023 USD)** | **Source** | **Country** | **Notes** |
| --- | --- | --- | --- | --- | --- |
| **Section A: Capital costs** | | | | | |
| **Capital costs: Medical equipment** | | | | | |
|  | X-ray machine (FUJIFILM FDR Smart) | 7,669 | Invoice | SA | 2021 price, uprated to 2023 using SA CPI; lifespan 11 years; 80% utilisation |
|  | X-ray machine (Delft Light) | 16,738 | Invoice | LS | 2020 price, uprated to 2023 using WB CPI; lifespan 6 years; 100% utilisation |
|  | Xpert MTB/RIF Ultra machine | 4,515 | Invoice | SA/LS | 2020/2021 price, uprated; lifespan 6 years; inclusive of laptop and software |
|  | CAD4TB box + software | 1,340 | Invoice | SA/LS | 2020/2021 price, uprated; lifespan 6 years |
|  | LumiraDx | 1,516 | Invoice | SA/LS | 2022/2023 price; lifespan 6 years; inclusive of scanner |
| **Capital costs: Vehicles** | | | | | |
|  | Vehicle fleet (2 IVECO Daily vans, 2 Ford Rangers, 2 caravans) | 6,680 | Invoice | SA | Annualised over lifespan; 80% utilisation; fuel and driver costs reported separately |
|  | Weekly vehicle rental (1 Toyota Fortuner, 1 Mercedes Sprinter Twin Cab) | 1,084 | Rental agreement | LS | Weekly hire inclusive of fuel, insurance, and driver. Treated as recurrent cost for LS. |
| **Capital costs: Other equipment (Lesotho only, total costs)** | | | | | |
|  | Tent | 379·68 | Invoice | LS | LSL 7,000/unit; 3 procured; used as temporary consultation rooms |
|  | Tables | 121·39 | Invoice | LS | LSL 2,238/unit; 4 procured |
|  | Chairs | 227·81 | Invoice | LS | LSL 4,200/unit; 10 procured |
|  | Heater | 27·12 | Invoice | LS | LSL 500/unit; 4 procured |
| **Section B: Recurrent costs** | | | | | |
| **Cost category** | **Item** | **Unit cost (2023 USD)** | **Source** | **Country** | **Notes** |
| **Recurrent costs: Fuel** | | | | | |
|  | Fuel cost per vehicle per month | 134·51 | Implementing partner records | SA | ZAR 2,480/month; 65% attributed to campaign (reflecting field day schedule); LS fuel costs included in vehicle hire |
| **Recurrent costs: Personnel** | | | | | |
|  | Enrolled nurse | 945·19 | National public healthcare sector salary scales | SA | ZAR 17,426/month; 80% LOE |
|  | Radiographer | 1,701·89 | National public healthcare sector salary scales | SA | ZAR 31,377/month; 80% LOE |
|  | POC device operator | 945·19 | National public healthcare sector salary scales | SA | ZAR 17,426/month; 80% LOE |
|  | Study driver / field support | 623·22 | National public healthcare sector salary scales | SA | ZAR 11,490/month; 80% LOE |
|  | Administrative and managerial staff | Varies | National public healthcare sector salary scales | SA | 2·5-20% LOE depending on role |
|  | Study nurse | 996·66 | National public healthcare sector salary scales | LS | LSL 18,375/month; 100% LOE |
|  | Nursing assistant | 626·47 | National public healthcare sector salary scales | LS | LSL 11,550/month; 100% LOE |
|  | Radiography assistant | 341·71 | National public healthcare sector salary scales | LS | LSL 6,300/month; 100% LOE |
|  | TB screener | 172·48 | National public healthcare sector salary scales | LS | LSL 3,180/month; 100% LOE |
|  | Administrative and managerial staff | Varies | National public healthcare sector salary scales | LS | 2·5–20% LOE; overnight allowances included for 5-week district deployment |
| **Recurrent costs: Screening costs** | | | | | |
|  | CAD4TBv7 imaging | 0·80 | Invoice | SA/LS | ZAR 14·75/unit (2023); uniform unit cost applied across both countries |
|  | HbA1c test kit | 3·84 | Invoice | SA/LS | ZAR 70·82/unit (2023); freight included |
|  | CRP test kit (LumiraDx) | 3·51 | Invoice | SA/LS | ZAR 59·94/unit (2023); freight included |
|  | Xpert MTB/RIF Ultra cartridge | 10·51 | Invoice | SA/LS | ZAR 184·00/unit (2023); freight included |
|  | NCD screening (HbA1c + BP) | 4·11 | Invoice | SA/LS | Composite cost per participant for NCD screening bundle |
|  | HIV rapid test - Alere Determine HIV ½ | 2·92 | Invoice | SA | ZAR 53·82/unit; includes freight |
|  | HIV confirmatory test - Uni-Gold Recombigen HIV 1/2 | 2·92 | Invoice | SA | ZAR 53·80/unit; includes freight |
|  | HIV rapid test - Alere Determine HIV 1/2 | 3·00 | Invoice | LS | ZAR 55·33/unit; includes freight |
|  | HIV confirmatory test - Uni-Gold Recombigen HIV 1/2 | 3·00 | Invoice | LS | ZAR 55·31/unit; includes freight |
| **Recurrent costs: Consumables** | | | | | |
|  | Sputum cups | 13·34 | Invoice | SA/LS | ZAR 245·99/pack; same cost both countries |
|  | Lancets | 12·50 | Invoice | SA | ZAR 230·49/pack |
|  | Lancets | 5·71 | Invoice | LS | LSL 105·30/pack |
|  | Alcohol swabs | 1·90 | Invoice | SA/LS | ZAR 35·04/pack; same cost both countries |
|  | X-ray gowns | 1·76 | Invoice | SA only | ZAR 32·50/pack; female participants only |
| **Capital costs: Other equipment (Lesotho only, total costs)** | | | | | |
|  | Tent | 379·68 | Invoice | LS | LSL 7,000/unit; 3 procured; used as temporary consultation rooms |
|  | Tables | 121·39 | Invoice | LS | LSL 2,238/unit; 4 procured |
|  | Chairs | 227·81 | Invoice | LS | LSL 4,200/unit; 10 procured |
|  | Heater | 27·12 | Invoice | LS | LSL 500/unit; 4 procured |
| **Currency conversion** | | | | | |
|  | ZAR / LSL to USD | 1 ZAR = 1 LSL = 0·05424 USD | World Bank / IMF | SA/LS | Mid-year 2023 market exchange rate. Pre-2023 costs uprated using World Bank CPI for South Africa and Lesotho respectively. |

*Notes: All equipment costs sourced from procurement invoices or supplier quotations. Pre-2023 costs uprated to 2023 prices using the World Bank Consumer Price Index for South Africa and Lesotho. Personnel costs based on national public healthcare sector salary scales, allocated by level of effort (LOE). Where unit costs were unavailable for one country, estimates were substituted from the other country. CRP: C-reactive protein; CAD: computer-aided detection; LOE: level of effort; CPI: consumer price index; BP: blood pressure; SA: South Africa; LS: Lesotho. For SectionA: Captial costs, the column headed ‘Annualised cost (2023 USD)’ reports the annualised equipment costs per year, not the one-time unit purchase price. Each value is the item’s purchase price up-rated to 2023 and annualised over its useful life, discounted at 3% in line with WHO-CHOICE guidelines. These annualised costs are derived from the unit purchase prices and used in the cost-per-case analysis.*

## Scenario analysis

Two pre-specified scenario analyses were conducted to assess the sensitivity of cost estimates to TB prevalence and screening volume. In Scenario 1, TB prevalence was modelled at 1%, a value consistent with published estimates for community-based screening in comparable high-burden settings (1). The number of participants and total costs were held constant at observed values; only the number of TB cases detected was recalculated based on the modelled prevalence, with the proportion of screen-positives receiving each test unchanged. In Scenario 2, screening volume was scaled to 100,000 participants at the observed TB prevalence. Country-specific participant numbers reflected the trial distribution (34,890 in South Africa and 65,110 in Lesotho, proportional to the trial ratio of approximately 35:65). Variable costs scaled proportionally with participant numbers; fixed costs were held constant. Cost parameters were sampled independently for each scenario in the probabilistic sensitivity analysis, such that total costs do not scale as a simple multiple across scenarios.

## Probabilistic sensitivity analysis

To account for parameter uncertainty in the cost estimates, a probabilistic sensitivity analysis (PSA) was conducted. Gamma distributions were assigned to primary cost categories including personnel, diagnostic tests (comprising the CRP and Xpert Ultra cartridge unit costs), consumables, and capital costs (vehicles and medical and diagnostic equipment), as well as to total screening volumes. Beta distributions were applied to observed TB prevalence and to the proportions of individuals receiving each screening test. Each parameter was assumed to vary within a 20% uncertainty range, in line with WHO-CHOICE recommendations (2). A total of 10,000 Monte Carlo simulations were run to generate mean estimates and 95% uncertainty intervals (UI) for total cost, average cost per participant screened, and average cost per TB case detected, reported for the primary analysis and both pre-specified scenario analyses. Cost parameters were sampled independently between the two screening approaches; no correlation structure was imposed across the CAD4TBv7 and CAD4TBv7-CRP cost distributions.

Since cost parameters are sampled independently for each scenario, total costs do not scale as a simple multiple across scenarios, in particular, scaling to 100,000 participants (Scenario 2) introduces additional sampling variation relative to the primary analysis. Empirical (deterministic) cost estimates were compared with the PSA-sampled means to ascertain consistency between the deterministic and the probabilistic estimates. In all cases, empirical estimates fell within the 95% UI of the PSA distribution. Across all cost categories and scenarios examined, the CAD4TBv7 approach retained the lower mean cost per TB case detected relative to the CAD4TBv7-CRP approach. All analyses were conducted in R version 4·4·1.

# Supplementary results

## Capital and recurrent costs

**Table G. Average value of capital and recurrent costs and their share of total costs by country and screening intervention (n=20,023)**

| **Country** | **Cost type** | **CAD4TBv7** | | **CAD4TBv7-CRP** | |
| --- | --- | --- | --- | --- | --- |
|  |  | TB | TB+HIV+NCD | TB | TB+HIV+NCD |
| Overall | Capital | 120,265 (30.6%) | 140,653 (23.5%) | 128,891 (28.7%) | 148,595 (22.7%) |
|  | Recurrent | 272,430 (69.4%) | 457,702 (76.5%) | 320,284 (71.3%) | 505,550 (77.3%) |
| South Africa | Capital | 48,111 (32.7%) | 65,477 (25.7%) | 55,233 (30.0%) | 72,209 (24.8%) |
|  | Recurrent | 99,103 (67.3%) | 188,929 (74.3%) | 129,092 (70.0%) | 218,571 (75.2%) |
| Lesotho | Capital | 72,154 (29.4%) | 75,176 (21.9%) | 73,658 (27.8%) | 76,386 (21.0%) |
|  | Recurrent | 173,327 (70.6%) | 268,773 (78.1%) | 191,192 (72.2%) | 286,979 (79.0%) |

Table G summarises the average capital and recurrent costs and their proportional contribution to total costs by country and screening intervention. Across both approaches and countries, recurrent costs accounted for the majority of total expenditure, representing 69-77% of overall costs. Capital costs comprised a larger share in South Africa than in Lesotho.

## Integrated screening costs

**Table H. Total costs and cost per TB case detected by screening approach and country - integrated screening (TB+HIV+NCD), primary analysis**

| **Outcome** | **CAD4TBv7 approach** | **CAD4TBv7-CRP approach** |
| --- | --- | --- |
| *Overall (n=20,023) \| Observed prevalence* | | |
| TB cases detected | 72 | 60 |
| TB diagnostic yield , per 1000 screened | 3.6 | 3.0 |
| HIV cases diagnosed | 61 | 61 |
| NCD cases screened positive (hypertension or diabetes)† | 3,404 | 3,404 |
| Total cost of screening, USD (95% UI) | 598,355 [470,731–743,389] | 654,145 [515,375–809,349] |
| Average cost per participant screened, USD (95% UI) | 30 [24–37] | 33 [26–40] |
| Average cost per TB case detected, USD (95% UI) | 8,310 [6,538–10,325] | 10,902 [8,590–13,489] |
| *South Africa (n=6,986) \| Observed prevalence* | | |
| TB cases detected | 31 | 29 |
| TB diagnostic yield , per 1000 screened | 4.4 | 4.2 |
| HIV cases diagnosed | 37 | 37 |
| NCD cases screened positive (hypertension or diabetes)† | 703 | 703 |
| Total cost of screening, USD (95% UI) | 254,406 [196,101–321,793] | 290,780 [223,717–368,016] |
| Average cost per participant screened, USD (95% UI) | 36 [28–46] | 42 [32–53] |
| Average cost per TB case detected, USD (95% UI) | 8,207 [6,326–10,380] | 10,027 [7,714–12,690] |
| *Lesotho (n=13,037) \| Observed prevalence* | | |
| TB cases detected | 41 | 31 |
| TB diagnostic yield , per 1000 screened | 3.1 | 2.4 |
| HIV cases diagnosed | 24 | 24 |
| NCD cases screened positive (hypertension or diabetes)† | 2,701 | 2,701 |
| Total cost of screening, USD (95% UI) | 343,949 [274,630–421,596] | 363,365 [291,658–441,333] |
| Average cost per participant screened, USD (95% UI) | 26 [21–32] | 28 [22–34] |
| Average cost per TB case detected, USD (95% UI) | 8,389 [6,698–10,283] | 11,721 [9,408–14,237] |

Note: Economic costs in 2023 USD. Mean costs presented with 95% uncertainty intervals (UI) from 10,000 Monte Carlo simulations. TB cases defined as Xpert MTB/RIF Ultra positive excluding trace results. HIV cases defined as newly diagnosed HIV-positive participants. NCD cases defined as participants screening positive for hypertension or diabetes. †NCD case counts are identical across both screening approaches as NCD screening does not differ by TB screening algorithm. TB-only costs are reported in the main text (Table 1).

Bringing the TB screening outcomes together, all 20,023 participants in the full analysis set (6,986 in South Africa and 13,037 in Lesotho) underwent CAD4TBv7 screening. Under the CAD4TBv7 approach, 4,491 participants screened positive and were indicated for confirmatory Xpert Ultra testing, of whom 4,409 had an available result, and 72 were diagnosed with TB, a diagnostic yield of 3.6 per 1,000 screened. Under the CAD4TBv7-CRP approach, 2,772 participants screened positive after CRP triage (either directly, at CAD scores above the upper threshold, or through a CRP result above 5 mg/L within the triage window), of whom 2,715 had an available Xpert Ultra result, and 60 were diagnosed with TB, a yield of 3.0 per 1,000 screened. Country-specific yields are shown above. The complete screening cascade for both approaches, including all intermediate result categories, is presented in the primary trial paper ((3) Fig B).

**Table I. Scenario analysis: total costs and cost per TB case detected by screening approach and country - integrated screening (TB+HIV+NCD)**

| **Outcome** | **CAD4TBv7 approach** | **CAD4TBv7-CRP approach** |
| --- | --- | --- |
| *Scenario 1 (Overall, n=20,023) \| 1% modelled TB prevalence* | | |
| TB cases detected | 136 | 113 |
| Total cost of screening, USD (95% UI) | 598,355 [470,731–743,389] | 654,145 [515,375–809,349] |
| Average cost per participant screened, USD (95% UI) | 30 [24–37] | 33 [26–40] |
| Average cost per TB case detected, USD (95% UI) | 4,400 [3,459–5,466] | 5,789 [4,561–7,164] |
| Scenario 2 (Overall, n=100,000) \| Observed prevalence | | |
| TB cases detected | 360 | 300 |
| Total cost of screening, USD (95% UI) | 2,773,317 [2,156,863–3,497,477] | 2,885,636 [2,248,459–3,616,082] |
| Average cost per participant screened, USD (95% UI) | 28 [22–35] | 29 [22–36] |
| Average cost per TB case detected, USD (95% UI) | 7,704 [5,991–9,715] | 9,619 [7,495–12,054] |
| *Scenario 2 (South Africa, n=34,890) \| Observed prevalence* | | |
| TB cases detected | 155 | 145 |
| Total cost of screening, USD (95% UI) | 1,401,303 [1,081,421–1,781,954] | 1,460,667 [1,126,324–1,853,906] |
| Average cost per participant screened, USD (95% UI) | 40 [31–51] | 42 [32–53] |
| Average cost per TB case detected, USD (95% UI) | 9,041 [6,977–11,496] | 10,074 [7,768–12,786] |
| *Scenario 2 (Lesotho, n=65,110) \| Observed prevalence* | | |
| TB cases detected | 205 | 155 |
| Total cost of screening, USD (95% UI) | 1,372,014 [1,075,442–1,715,523] | 1,424,969 [1,122,135–1,762,176] |
| Average cost per participant screened, USD (95% UI) | 21 [17–26] | 22 [17–27] |
| Average cost per TB case detected, USD (95% UI) | 6,693 [5,246–8,368] | 9,193 [7,240–11,369] |

Note: Economic costs in 2023 USD. Mean costs presented with 95% uncertainty intervals (UI) from 10,000 Monte Carlo simulations. Scenario 1: TB prevalence modelled at 1% with participant numbers and total costs held constant at observed values. Scenario 2: screening volume scaled to 100,000 participants at observed prevalence, with country proportions reflecting the trial distribution. TB-only scenario costs are reported in the main text (Table 2).

The marginal cost of extending TB-only to integrated screening was estimated by dividing the difference in total costs between integrated and TB-only screening by the number of additional HIV or NCD positive screens identified. Across both approaches combined, the total additional cost of integrated screening was USD 205,660 for the CAD4TBv7 approach, spread across 3,465 additional HIV or NCD positive screens, yielding an average marginal cost of approximately USD 59 per additional HIV or NCD case detected.

**Table J. Total and mean cost per TB, NCD, or HIV case observed by country and intervention arm**

| Outcome | **CAD4TBv7** | **CAD4TBv7-CRP** |
| --- | --- | --- |
|  | TB+HIV+NCD | TB+HIV+NCD |
| *Overall (n=20,023) \| Observed prevalence* | | |
| TB cases detected | 72 | 60 |
| NCD cases screened positive(diabetes or hypertension) | 3404 | 3404 |
| HIV cases diagnosed | 61 | 61 |
| TB, NCD, or HIV case observed | 3537 | 3525 |
| Total cost of screening | 598,355 [470,731 – 743,389] | 654,145 [515,375 – 809,349] |
| Cost per TB, NCD, or HIV case observed | 169 [133 - 210] | 185 [146 - 230] |
| *South Africa (n=6,986) \| Observed prevalence* | | |
| TB cases detected | 31 | 29 |
| NCD cases screened positive | 703 | 703 |
| HIV cases diagnosed | 37 | 37 |
| TB, NCD, or HIV case observed | 771 | 769 |
| Total cost of screening | 254,406 [196,101 – 321,793] | 290,780 [223,717 – 368,016] |
| Cost per TB, NCD, or HIV case observed | 330 [254 - 417] | 378 [291 - 479] |
| *Lesotho (n=13,037) \| Observed prevalence* | | |
| TB cases detected | 41 | 31 |
| NCD cases screened positive | 2701 | 2701 |
| HIV cases diagnosed | 24 | 24 |
| TB, NCD, or HIV case observed | 2766 | 2756 |
| Total cost of screening | 343,949 [274,630 – 421,596] | 363,365 [291,658 – 441,333] |
| Cost per TB, NCD, or HIV case observed | 124 [99 - 152] | 132 [106 - 160] |

Table J summarises the total and mean costs per TB, NCD, or HIV case detected across countries and intervention arms. When combining all screening outcomes, the overall cost per case observed was USD 169 (95% UI: 133–210) for the *CAD4TBv7* approach and USD 185 (146–230) for *CAD4TBv7-CRP*. South Africa showed higher costs per case detected (USD 330–378) compared with Lesotho (USD 124–132), primarily due to the substantially lower number of NCD cases screened positive.

## Cost drivers

**Fig A. Drivers of overall costs by approach and country for screening TB**

**Fig B. Drivers of overall costs by approach and country for screening TB, HIV, and NCD**

## Financial costs

**Table K. Financial cost outcomes by approach, delivery, and country**

| Outcome | **CAD4TBv7** | | **CAD4TBv7-CRP** | |
| --- | --- | --- | --- | --- |
|  | TB only | TB+HIV+NCD | TB only | TB+HIV+NCD |
| Overall | | | | |
| Total cost of screening | 388,598 | 575,987 | 434,597 | 628,691 |
| Cost per participant screened | 19 | 29 | 22 | 31 |
| Cost per TB case detected | 5,397 | 8,000 | 7,243 | 10,478 |
| South Africa | | | | |
| Total cost of screening | 150,527 | 241,541 | 178,897 | 276,616 |
| Cost per participant screened | 22 | 35 | 26 | 40 |
| Cost per TB case detected | 4,856 | 7,792 | 6,169 | 9,538 |
| Lesotho | | | | |
| Total cost of screening | 238,071 | 334,447 | 255,699 | 352,075 |
| Cost per participant screened | 18 | 26 | 20 | 27 |
| Cost per TB case detected | 5,807 | 8,157 | 8,248 | 11,357 |

Table K compares the total and unit financial costs of the CAD4TBv7 and CAD4TBv7-CRP screening approaches across interventions and countries. Overall, the combined TB+HIV+NCD screening approach was more costly than TB-only screening, largely due to additional consumables and personnel time. The CAD4TBv7-CRP approach consistently incurred higher total and per-participant costs than CAD4TBv7 alone. The cost per TB case detected ranged from USD 4,856 in South Africa (CAD4TBv7, TB-only) to USD 11,357 in Lesotho (CAD4TBv7-CRP, TB+HIV+NCD), reflecting variation in disease prevalence and operational context between countries.

## Scenario analysis

**Table L. Total costs and cost per screening and TB case detected when administering Xpert Ultra for all under different scenarios (post-hoc)**

|  | Base scenario(n=20,023) \| Observed prevalence | Scenario 1 (n=20,023) \| 1% modelled prevalence | Scenario 2 (n=100,000) \| Observed prevalence |
| --- | --- | --- | --- |
| TB cases detected | 85 | 160 | 410 |
| Total cost of screening | 570,807 [436,484 – 731,441] | 570,807 [436,484 – 731,441] | 2,764,505 [2,071,968 – 3,572,389] |
| Cost per participant screened | 29 [22 – 37] | 29 [22 – 37] | 28 [21 – 36] |
| Cost per TB case detected | 6,961 [5,323 – 8,920] | 3,568 [2,728 – 4,572] | 6,743 [5,054 – 8,713] |

Table L presents costs under a post-hoc supplementary scenario in which all participants were assumed to undergo confirmatory Xpert MTB/RIF Ultra testing regardless of CAD4TBv7 score. This scenario was included to contextualise the trial screening approaches against a higher-yield but more resource-intensive alternative, and should be interpreted as exploratory. Total screening costs ranged from USD 0.57 million in the base case (20,023 participants) to USD 2.76 million when scaled to 100,000 participants. The cost per participant screened remained relatively stable across scenarios (USD 28–29), while the cost per TB case detected declined markedly with increasing prevalence: from USD 6,961 in the base case to USD 3,568 under a modelled 1% prevalence.

## Sensitivity analysis

**Fig C. Probabilistic sensitivity analysis of total screening costs by intervention arm and country**
*Note: The horizontal bar charts represent the mean of the sampled costs, and the black lines represent the 95% uncertainty range around the average sampled cost. The black dots are the calculated estimates of the unsampled empirical costs. Panel A shows combined results for both countries, Panel B for South Africa, and Panel C for Lesotho.*

**Table M. Cost per TB case detected under alternative case denominators**

| Denominator set | CAD4TBv7 cost per case detected | CAD4TBv7-CRP cost per case detected | Difference |
| --- | --- | --- | --- |
| Full analysis set (72 vs 60) | 5,454 | 7,486 | 37.3% |
| Primary outcome set (69 vs 60) | 5,691 | 7,486 | 31.5% |

Note: Economic costs in 2023 USD. Total screening costs are held constant, only the number of TB cases detected differs between the full analysis set and the primary outcomes set. The CAD4TBv7-CRP count is identical across sets.

# References

1.	Scott AJ, Perumal T, Pooran A, Oelofse S, Jaumdally S, Swanepoel J, et al. Clinical evaluation of computer-aided digital x-ray detection of pulmonary tuberculosis during community-based screening or active case-finding: a case–control study. Lancet Glob Health. 2025 Mar 1;13(3):e517–27. doi:10.1016/S2214-109X(24)00516-3 PubMed PMID: 40021309.

2.	World Health Organization. WHO-CHOICE [Internet]. Geneva; 2014. Available from: https://www.who.int/news-room/questions-and-answers/item/who-choice-frequently-asked-questions

3.	Reither K, Vanobberghen F, Verjans A, Harkare HV, Jacobs BKM, Heerden A van, et al. Community-based tuberculosis screening with computer-aided detection technology alone and in combination with point-of-care C-reactive protein testing: a paired screen-positive trial. Lancet Infect Dis. 2026 Jul 1;0(0). doi:10.1016/S1473-3099(26)00222-7 PubMed PMID: 42385764.
