# Supplementary material for: Comparative cost analysis of community-based tuberculosis screening: Computer-Aided Detection (CAD) alone versus CAD with point-of-care C-reactive protein testing in South Africa and Lesotho
Source: PLOS Glob Public Health. 2026 Sep 21;6(9):e0007341. doi: 10.1371/journal.pgph.0007341 (PMC13592630; doi:10.1371/journal.pgph.0007341)
Supplement: Supplementary file 2 [file pgph.0007341.s003.docx]

**Subject: Response to Reviewers for submission ID: PGPH-D-26-02457**

Dear Dr. Zwerling,

Many thanks for the opportunity to revise our manuscript titled “Comparative cost analysis of community-based tuberculosis screening: Computer-Aided Detection (CAD) alone versus CAD with point-of-care C-reactive protein testing in South Africa and Lesotho”. We are thankful for the opportunity to review and strengthen our manuscript.

We are grateful to both reviewers for a careful and constructive assessment. Their comments have improved the manuscript, and we have made all revisions with the focus of the paper in mind: the comparative cost analysis of community-based CAD4TBv7 screening alone versus CAD4TBv7 combined with point-of-care C-reactive protein testing.

The parent trial has since been published and is now cited throughout as reference #9 (Reither et al., 2026). We have clarified how the present analysis extends that work, namely by separating capital from recurrent costs, identifying the dominant cost drivers in each country, reporting financial alongside economic costs, costing the integrated HIV and NCD screening delivered in the same campaign, and providing the unit costs and code needed to reproduce and transfer the estimates. We have made the paired within-person design explicit in the Methods, standardised terminology with the supplementary tables (including "triage window" and the screen-negative lower tier), and expanded the description of how economic and opportunity costs were measured and valued.

In response to specific points, we have added a sensitivity analysis using denominators held consistent with the primary trial, expanded the probabilistic sensitivity analysis, reported TB diagnostic yield per 1,000 screened, and extended the Discussion to address integrated screening, the distinction between our community ACF setting and the clinic-based PLHIV population underlying the WHO CRP recommendation, the treatment of Xpert trace results, and the policy framing of vertical active case finding. We have also taken this opportunity to make a minor correction to Table S6 and have added a new supplementary table (Table S5) on procurement costs, prompted by interest from organisations working on the scale-up of this approach.

We have provided a clean manuscript and a version with tracked changes, alongside a point-by-point response below. We look forward to any further feedback you may have.

Kind regards,

Harsh Vivek Harkare, on behalf of all co-authors

------------------------------------------------------------------------------------------

## Journal requirements

1. Inclusivity in global research questionnaire.

- Completed and uploaded as Supporting Information.

2. Code sharing. Author-generated code underpinning the findings must be made available without restriction on publication, shared in a way that supports reproducibility and reuse.

- All author-generated analysis code (R scripts) underlying the findings have been deposited without restriction in a public repository and are openly available at <https://github.com/harshvh/tbtriage-cost-analysis>

3. Financial Disclosure statement. Provide a detailed statement in full sentences with the exact wording for publication: (a) state the initials of each author receiving each grant alongside each funding source; and (b) state the funders' role, or that they had no role in study design, data collection and analysis, decision to publish, or preparation of the manuscript.

- Amended in the submission system. The Financial Disclosure field now reads: "This work was supported by the European and Developing Countries Clinical Trials Partnership (EDCTP2; grant RIA2018D-2498 to KR), which provided salary support. The funders had no role in study design, data collection and analysis, decision to publish, or preparation of the manuscript." The same funder and grant number are entered in the Funding Information tab.

4. Consistency of funding information. Ensure the funders and grant numbers match, and are listed in the same order, between the Financial Disclosure field and the Funding Information tab.

- We have confirmed that the funder (EDCTP2) and grant number (RIA2018D-2498) are entered identically, and in the same order, in both the Financial Disclosure field and the Funding Information tab.

5. Competing interests statement. Update the online statement (e.g. "The authors have declared that no competing interests exist").

- We have updated the online Competing Interests statement to read: “The authors have declared that no competing interests exist.”

6. Data availability. Data indicated as available on acceptance must be made freely accessible on publication, except where deposition would breach the ethics-approved protocol. If unable to share fully, revise the statement to explain the reasoning for the editor's consideration of an exemption.

- We have provided a data-availability statement describing controlled access to a de-identified dataset, given the sensitivity of individual-level socioeconomic and disease data in a small two-district sample. This is the same data sharing policy as the manuscript of the parent trial published in the Lancet Infectious Disease, as we are unable to freely share the full dataset publicly due to ethical constraints. The pseudo-anonymized dataset is available at Zenodo (Reither, K., et al. ‘Community-based Tuberculosis Screening Using Computer-Aided Detection (CAD) Technology Alone Compared with CAD Combined with Point-of-care C-reactive Protein Testing: A Paired Screen-positive Trial’. Zenodo, 17 Mar. 2026, <https://doi.org/10.5281/zenodo.19068197>).

7. Figures. Provide separate main figure files in .tif or .eps format only, each under 10 MB, remove figures embedded in the manuscript file (retaining the captions), and process files through PLOS's NAAS tool.

- The main-text figure (Figure 1) has been supplied as a separate high-resolution file in .tif format, under 10 MB, and processed through the NAAS tool; it has been removed from the manuscript file with its caption retained. Supplementary figures are provided within the Supplement.

8. Supporting Information. Add a full list of SI legends before or after the reference list.

- A full list of Supplementary Information legends has been added after the reference list.

9. Reviewer-recommended citations. Evaluate any suggested works for relevance; there is no requirement to cite them unless the editor indicates otherwise.

- No additional publications were explicitly recommended by the reviewers for citation.

## Reviewer #1

**1**. Introduction, lines 90-93. The programmatic costing perspective used here differs from the study already in press. Please explain more clearly how the current analysis differs from that earlier work and what additional public-health or policy value it provides, as the justification for a separate analysis is not entirely clear. The cited study could also not be located in the reference list, so please check that the reference has been included.

- We thank the reviewer. On the reference: the parent trial was in press at submission and its citation was incomplete. It is now published and cited throughout as reference #9 (Reither et al., 2026).
- On the case for a separate analysis: the parent trial reported cost per TB case detected, total cost, and cost per participant screened as headline co-primary outcomes, and concluded that the *CAD4TBv7-CRP approach* was dominated. It did not take those figures further. It did not separate capital from recurrent costs, identify the cost drivers in each country, report financial alongside economic costs, cost the HIV and NCD screening delivered in the same campaign, or give the unit costs and assumptions needed to reproduce or transfer the estimates. This paper provides that detail.
- On the policy value: the parent trail only reports the headline cost per case, which tells a programme manager what the campaign cost, not where the money went or how to adapt it. Here we give the cost structure and the dominant drivers in each country, so programmes can budget for community ACF and match algorithm and delivery choices to their own cost profile. We also cost the integrated campaign: roughly USD 59 per additional HIV or NCD case detected, and USD 169 per positive screen across all three conditions, which cover the integrated screening angle the parent trial did not cover. The full unit-cost inputs and analysis code also make the estimates reproducible.
- We have added a line to the Introduction to clarify what the parent trial reported and what this manuscript reports (p. 4).

2. Did the authors consider a third strategy based on sequential screening in a specific high-risk group, such as people living with HIV, given prior evidence that CRP may perform reasonably well as a TB screening tool in this population? This is discussed at lines 300-309; is it possible to conduct this analysis with the available data?

- Thank you for flagging this. We did consider this strategy and yes, the analysis is feasible with our data and was pre-specified and already carried out in the parent trial paper (Reither et al. 2026, Supplementary Table 3b). We saw no effect modification of TB case yield by HIV status: among the 20 PLHIV with TB, the *CAD4TBv7-CRP approach* detected fewer cases than *CAD4TBv7* alone (16 vs 18; difference -0.10, 95% CI -0.34 to 0.14). Because CRP-based screening detected fewer cases even within PLHIV, restricting it to that group would not have improved case detection in this community setting, so a separate PLHIV-targeted cost analysis would not change the conclusion. We have added this finding to the Discussion (p. 16). The related question of cost within the PLHIV subgroup, and why the community PLHIV population here differs from the clinic-based PLHIV group for whom WHO recommends CRP, is addressed in our response to Reviewer 2, point 5.

3. The scenario analysis could be strengthened by varying some of the main cost inputs, for example a lower CRP reagent price, or vehicle hire versus one-off procurement. This may help readers understand the circumstances under which CRP-based screening could become more affordable or cost-effective.

- Thank you for the interesting point. We would first like to note that our comparative cost analysis does not depend on these cost inputs. This is because the *CAD4TBv7-CRP approach* detected fewer TB cases than *CAD4TBv7* (60 vs 72) and is dominated on cost per case regardless of unit costs.
- The primary lever determining cost-effectiveness in our study context is case yield, not unit price. Consequently, lowering the CRP reagent price, vehicle hire, or any other single cost item can reduce absolute cost but cannot make the *CAD4TBv7-CRP approach* cost-effective unless it also averts more cases.
- We present here a quick arithmetic exercise to demonstrate our point, since it is not presented anywhere else: For the *CAD4TBv7-CRP approach* to match the cost per case of the *CAD4TBv7 approach* (USD 5,454), its total cost would need to fall to 5,454 × 60 = USD 327,240. Its actual total cost is USD 449,175, so the required reduction of USD 121,935 exceeds the entire CRP testing expenditure.
- For the effect of varying individual cost items, the probabilistic sensitivity analysis already covers this. We varied all primary cost categories, including the CRP and Xpert unit costs and capital (vehicles and equipment), by +/-20%, and found the *CAD4TBv7 approach* to be dominant throughout. We have expanded the description of this analysis accordingly in the Supplement (p. 16).
- The circumstances under which CRP-based sequential screening could become more favourable are therefore governed by CRP specificity and the underlying comorbidity burden rather than by unit costs, as noted in the Discussion (p. 16).

4. Please consider adding a table that brings together the main results for integrated screening: the number screened, the number who screened positive, the number receiving confirmatory testing, the number of TB cases detected, and the overall diagnostic yield. Presenting these in one place would ease reading of the results and discussion.

- Thank you for the comment. The main integrated screening results are already consolidated in the Supplement. Supplementary Tables 8 reports, for each approach and country, the number screened and the TB, HIV, and NCD cases detected, together with total cost, cost per participant screened, and cost per TB case detected. Supplementary Table 10 adds the combined cost per case across all three conditions, and Supplementary Table 9 provides the scenario analyses.
- The detailed TB screening cascade, including the numbers screening positive and receiving confirmatory Xpert Ultra testing, is presented in the primary trial paper (Reither et al. 2026, Supplementary figure 2), which we now cite explicitly for the readers to locate.
- To completely address your comment, we have added the TB diagnostic yield per 1,000 screened to Supplementary Table 8 (overall 3.6 for CAD4TBv7 and 3.0 for CAD4TBv7-CRP; South Africa 4.4 and 4.2; Lesotho 3.1 and 2.4). Finally, we have added a brief paragraph describing these results and presenting all information in one place while avoiding duplication of the cascade figure already published in the companion paper (p. 18 of the Supplement).

5. The calculation of economic costs needs to be described in more detail. The manuscript refers to opportunity costs, but it is not clear how these were measured or valued.

- We have expanded the description of the economic cost calculation in the Supplement accordingly (p. 9). In short, economic costs reflect the opportunity cost of resources and differ from financial costs in that value-added tax (15%) is excluded as a transfer payment, and capital items (equipment and vehicles) are valued at their equivalent annual cost, obtained by annualising the VAT-exclusive, CPI-adjusted purchase price over each item's useful life at a 3% discount rate and weighting by the proportion allocated to the campaign. This discounted annualisation constitutes the opportunity-cost valuation of capital. Personnel were valued at full salary by level of effort, and recurrent items at the prices paid. We hope this clarifies how opportunity costs were measured and valued.

6. Were the same participants used in both study arms? If the populations differed, comparing the absolute number of TB cases detected may be misleading, because the arm with more participants, or a population with higher underlying TB risk, may naturally identify more cases. In addition to absolute numbers, please present percentages, diagnostic yield, or cases detected per 1,000 people screened to allow a fairer comparison.

- We are glad to clarify. The TB TRIAGE+ trial used a paired screen-positive design (within-person comparison), in which every participant was assessed under both screening approaches. The two arms therefore comprise the same 20,023 individuals, not separate populations, and are identical in size and underlying TB risk. The difference in TB cases detected (72 under CAD4TBv7 vs 60 under CAD4TBv7-CRP) reflects the two algorithms applied to the same people, so the absolute counts are directly comparable and cannot be explained by differences in population size or risk. We have made the paired within-person design explicit in the Methods (p. 5). As per your suggestion, we now also report the diagnostic yield per 1,000 screened alongside the absolute counts, added to Supplementary Table 8 and in text immediately following it (Supplement p. 18).

## Reviewer #2

1. Clarity and structure of screening approaches (lines 109-119). (a) The standalone CAD4TBv7 description reports outcomes for all participants, but the combined CAD4TBv7-CRP description specifies only the intermediate and upper scoring tiers and does not state the workflow or outcome for the lowest tier (≤5 in South Africa, ≤10 in Lesotho; Supplementary Table 1). For parallel structure, please state explicitly in the main text that these lower-tier participants were considered screen-negative. (b) Please replace the generic term "window" with "triage window" to align the main text with the supplementary tables.

- Thank you for the pointer.
- (a) We have revised the description of the *CAD4TBv7-CRP approach* to state explicitly that participants with a CAD4TBv7 score at or below the lower threshold (≤5 in South Africa, ≤10 in Lesotho) were considered screen-negative and received no further testing, similar to the CAD4TBv7 alone description (p. 6).
- (b) We have replaced "window" with "triage window" in the main text methods to align with the terminology used in the supplementary tables.

2. Choice of denominator in the Full Analysis Set. The authors report 72 TB cases for the CAD4TBv7 approach in the FAS versus 69 in the primary trial analysis; the three additional cases were excluded from the primary analysis for missing data in the alternative track, and including them reduces the average cost per case in the CAD arm by roughly 4%. Please provide a sensitivity analysis showing cost-per-case outcomes with denominators held strictly consistent with the primary trial (n=69 vs n=60), to clarify whether this choice over-represented the cost-efficiency of the CAD-only approach.

- As requested, we have added a sensitivity analysis using denominators held consistent with the primary trial outcome set (69 for *CAD4TBv7* and 60 for *CAD4TBv7-CRP*). Holding total costs constant, the average cost per TB case detected becomes USD 5,691 for *CAD4TBv7* (from USD 5,454) and remains USD 7,486 for *CAD4TBv7-CRP*. The difference narrows from 37.3% to 31.5%, but *CAD4TBv7* still remains less costly per case and *CAD4TBv7-CRP* remains dominated, as it continues to detect fewer cases (69 vs 60).
- We note that the full analysis set is the appropriate primary denominator for a cost analysis, because it keeps the numerator (costs incurred) and denominator (cases detected) on the same population. The three additional cases detected under the *CAD4TBv7 approach* are genuine detections whose screening costs are included in the total, so excluding them from the denominator alone would understate rather than overstate the cost-efficiency of CAD4TBv7. The primary-set result is therefore presented as a sensitivity analysis (Supplementary Table 13), and it does not alter the conclusion.

3. Workforce models and task-shifting. Personnel was the dominant cost driver in South Africa (up to 58.8% of expenditure), and the average cost per participant screened was higher in South Africa than Lesotho ($21 vs $19), suggesting a high fixed-cost burden from a senior nursing cadre. Would the authors consider modelling a hypothetical task-shifting scenario, specifically whether adopting a junior nursing-assistant model would alter the fiscal viability of the CAD4TBv7-CRP track in South Africa?

- We thank the reviewer for this suggestion. Personnel is largely a shared field-team cost, meaning that the same team delivers both approaches in each country. The small difference between the two screening approaches comes from the additional test step in the *CAD4TBv7-CRP approach*, which is the more staff-intensive of the two. However, a lower-cost staffing model would reduce personnel in both approaches, with a marginally larger absolute saving for *CAD4TBv7-CR*P since it has a larger staffing requirement. But this would not change the number of TB cases detected. Since *CAD4TBv7-CRP* *approach* detects fewer cases (60 vs 72), it remains dominated on cost per case under any staffing model. Task-shifting could change the absolute affordability of community ACF, which applies to both approaches, but not the comparison which is the primary focus of the manuscript. We have therefore not added it as a formal scenario.

4. Policy implications and sustainability of vertical ACF. Scenario 2 shows that the average cost per TB case detected did not decrease meaningfully with scale, because recurrent costs (67-79%) scale proportionally with volume. Given this flat economies-of-scale effect, the authors are encouraged to expand the discussion with explicit policy guidance: at an observed prevalence of 0.3%, should vertical ACF programmes be deprioritised in favour of integrated screening models, which reduced the cost per case detected from $5,454 to $169?

- We agree this is an important policy question and have expanded the Discussion accordingly (p. 17 and p. 18).
- We would first clarify a distinction in denominators. The USD 5,454 figure is the average cost per TB case detected under TB-only screening, whereas the USD 169 figure is the average cost per positive screen across TB, HIV, and NCD combined under integrated screening. These are not the same output. Integrated screening does not detect TB cases more cheaply, but rather it detects the same TB cases at a modestly higher total, while also identifying HIV and NCD cases on the same infrastructure, which lowers the cost-per-positive screen but not the cost per TB case. Integrated screening is therefore not a substitute for community ACF but could rather be a more efficient way of delivering it, and the two figures cannot be read as a reduction in the cost per TB case detected.
- We have added a point on the prevalence-conditional efficiency gains from integrated TB screening (p. 16), and that can be greatest at low TB prevalence. TB-only screening is costly per case and does not become cheaper with scale.
- Finally, we have also added a statement on the case for prioritisation of vertical ACF. Namely, that this issue cannot be answered by a cost analysis alone since it depends on the value placed on TB case detection relative to averted transmission and downstream health outcomes. We have flagged this as a priority for future cost-effectiveness analysis linking these cost estimates to health outcomes in the Conclusion.

5. Economic performance of CRP in PLHIV subpopulations. (a) WHO recommends POC-CRP specifically as a TB screening tool among people living with HIV, yet the combined CAD4TBv7-CRP approach was 37% more expensive per case detected than standalone CAD4TBv7 in the general community population. (b) It is worth exploring whether this cost-inefficiency is driven by a dilution effect from screening a broad, unselected population. (c) Please provide a stratified sub-analysis or expanded discussion comparing the cost of the CAD4TBv7-CRP approach within the PLHIV subgroup versus the general population, to inform programmes evaluating CRP triage within HIV clinics rather than community-based ACF.

- We thank the reviewer, and agree it is important to separate the community setting studied here from the clinical setting in which WHO recommends POC-CRP. WHO recommends CRP for people already known to be living with HIV and attending HIV care, where TB prevalence is high and CRP is applied at entry to care. Our trial is community-based active case finding in an unselected population whose HIV status is not known at screening entry, so CRP cannot be targeted to people living with HIV in this model.
- On the dilution point: the 37% figure is a within-population comparison, since both approaches are applied to the same participants. Population size differences can shift the absolute cost per case under either approach, but not the relative penalty of adding CRP. The stratified yield analysis in the parent trial illustrates this explicitly: within the PLHIV subgroup, the *CAD4TBv7-CRP approach* detected fewer cases than *CAD4TBv7* alone (16 vs 18 of 20; difference -0.10, 95% CI -0.34 to 0.14). Specifically, it missed 4 cases that *CAD4TBv7* identified while detecting 2 cases that *CAD4TBv7* missed (Reither et al., 2026 – Supplementary Table 3b). So the ‘disadvantage’ of CRP triage is already present among PLHIV in our study setting, and it is not an artefact of screening a broad population.
- On the cost point: a stratified cost-per-case sub-analysis by HIV status is unfortunately not informative with our data. The costing is bottom-up and campaign-level: chest X-ray, CAD, mobile teams, and personnel are shared across all participants regardless of HIV status and cannot be attributed to the PLHIV subgroup in a meaningful manner. Since CRP adds cost while detecting fewer cases within the PLHIV subgroup, restricting it to people living with HIV would not make it more cost-efficient than *CAD4TBv7* alone in this setting. Our findings therefore do not contradict the WHO recommendation; they address a different population, entry point, and comparator. The related yield question is addressed in our response to Reviewer 1, point 2.

6. Clinical utility and impact of Xpert "trace" results. Active TB cases were defined by excluding Xpert trace results, but in high HIV co-infection settings trace results often carry clinical weight and may represent early-stage paucibacillary disease. Could the authors discuss or model how including trace results in the "cases detected" denominator would affect the analysis, and whether this would narrow or widen the 37% cost disparity between the two algorithms?

- We thank the reviewer and agree that trace results can carry clinical weight in these settings. In the parent trial, 21 participants with a trace result were referred and subsequently tested Xpert-positive at a health facility (Supplementary Table 2 of the parent trial paper) (Reither et al., 2026).
- The parent trial pre-specified two sensitivity analyses counting trace as a case. Under both, yield remained higher for the *CAD4TBv7 approach* and the difference remained statistically significant: 97 versus 84 when trace was counted for all participants, and 84 versus 72 when restricted to those without a prior TB history (Reither et al., 2026). The case definition therefore changes neither the direction nor the significance of the yield gap.
- For the cost analysis, including trace would change only the denominator, since these participants had already undergone Xpert Ultra and their screening costs are captured in each approach's total. Under the paired screen-positive design, every trace case detected under *CAD4TBv7-CRP* is also detected under *CAD4TBv7*, so the *CAD4TBv7* denominator gains at least as many cases as the *CAD4TBv7-CRP* denominator. The ordering of the two approaches in terms of the cost per TB case detected is thus invariant to the case definition, and *CAD4TBv7-CRP* remains dominated under any trace-inclusive denominator. We have noted this in the Discussion (p. 17).

We believe these revisions have sharpened the paper's focus and addressed the reviewer's concerns, and we sincerely thank both reviewers again for feedback that materially improved the manuscript.

Sincerely,

Harsh Vivek Harkare, on behalf of all co-authors

## References

Reither, K., Vanobberghen, F., Verjans, A., Harkare, H. V., Jacobs, B. K. M., Heerden, A. van, Misra, S., Tediosi, F., Brugger, C., Labhardt, N. D., Kamele, M., Keitseng, M., Madonsela, T., Kurscheid, J., Muhairwe, J., Keter, A. K., Murphy, K., Ginneken, B. van, Gils, T., … Ayakaka, I. (2026). Community-based tuberculosis screening with computer-aided detection technology alone and in combination with point-of-care C-reactive protein testing: A paired screen-positive trial. *The Lancet Infectious Diseases*, *0*(0). https://doi.org/10.1016/S1473-3099(26)00222-7
