# Supplementary material for: Comparative cost analysis of community-based tuberculosis screening: Computer-Aided Detection (CAD) alone versus CAD with point-of-care C-reactive protein testing in South Africa and Lesotho
Source: PLOS Glob Public Health. 2026 Sep 21;6(9):e0007341. doi: 10.1371/journal.pgph.0007341 (PMC13592630; doi:10.1371/journal.pgph.0007341)
Supplement: Peer review history

# Peer review: Decision Letter 0

**DOI:** 10.1371/journal.pgph.0007341.r001

**Authors:** Alice Zwerling

30 Jul 2026

PGPH-D-26-02457

Comparative cost analysis of community-based tuberculosis screening: Computer-Aided Detection (CAD) alone versus CAD with point-of-care C-reactive protein testing in South Africa and Lesotho

PLOS Global Public Health

Dear Dr. Harkare,

Thank you for submitting your manuscript to PLOS Global Public Health. After careful consideration, we feel that it has merit but does not fully meet PLOS Global Public Health’s publication criteria as it currently stands. Therefore, we invite you to submit a revised version of the manuscript that addresses the points raised during the review process.

Please submit your revised manuscript by Sep 28 2026 11:59PM. If you will need more time than this to complete your revisions, please reply to this message or contact the journal office at globalpubhealth@plos.org. When you're ready to submit your revision, log on to https://www.editorialmanager.com/pgph/ and select the 'Submissions Needing Revision' folder to locate your manuscript file.

Please include the following items when submitting your revised manuscript:

- A letter that responds to each point raised by the editor and reviewer(s). You should upload this letter as a separate file labeled 'Response to Reviewers'.
- A marked-up copy of your manuscript that highlights changes made to the original version. You should upload this as a separate file labeled 'Revised Manuscript with Track Changes'.
- An unmarked version of your revised paper without tracked changes. You should upload this as a separate file labeled 'Manuscript'.

Guidelines for resubmitting your figure files are available below the reviewer comments at the end of this letter.

As the corresponding author, your ORCID iD is verified in the submission system and will appear in the published article. PLOS supports the use of ORCID, and we encourage all coauthors to register for an ORCID iD and use it as well. Please encourage your coauthors to verify their ORCID iD within the submission system before final acceptance, as unverified ORCID iDs will not appear in the published article. *Only*  the individual author can complete the verification step; PLOS staff *cannot*  verify ORCID iDs on behalf of authors.

We look forward to receiving your revised manuscript.

Kind regards,

Alice Zwerling, PhD

Academic Editor

PLOS Global Public Health

Journal Requirements:

Additional Editor Comments (if provided):

[Note: HTML markup is below. Please do not edit.]

Reviewers' comments:

Reviewer's Responses to Questions

**Comments to the Author**

1. Does this manuscript meet PLOS Global Public Health’s publication criteria?

Reviewer #1: Yes

Reviewer #2: Yes

**********

2. Has the statistical analysis been performed appropriately and rigorously?

Reviewer #1: Yes

Reviewer #2: Yes

**********

3. Have the authors made all data underlying the findings in their manuscript fully available (please refer to the Data Availability Statement at the start of the manuscript PDF file)?

The PLOS Data policy

Reviewer #1: Yes

Reviewer #2: Yes

**********

4. Is the manuscript presented in an intelligible fashion and written in standard English?

Reviewer #1: Yes

Reviewer #2: Yes

**********

Reviewer #1: 1. In the Introduction, particularly lines 90-93, the authors mention that the costing perspective(programmatic) used in this study differs from that of a study that is already in press . It would be helpful to explain more clearly how the current analysis differs from the earlier work and what additional public health or policy value it provides. At present, the justification for conducting a separate analysis is not entirely clear. I was also unable to locate the cited study in the reference list, so please check that the reference has been included.

2. Did the authors consider including a third strategy based on sequential screening in a specific high-risk group, such as people living with HIV? Previous evidence suggests that CRP may perform reasonably well as a TB screening tool in this population. It is great that you have discussed this in lines 300-309, is it possible to do this analysis with the data you have?

3. The scenario analysis could be strengthened by varying some of the main cost inputs. For example, how would the results change if the price of CRP reagents were lower? Or looking at the vehicle hire vs once of procurement.This may help readers understand the circumstances under which CRP-based screening could become more affordable or cost-effective.

4. Please consider adding a table that brings together the main results for integrated screening. This could include the number screened, the number who screened positive, the number receiving confirmatory testing, the number of TB cases detected, and the overall diagnostic yield. Presenting these results in one place would make the reading of the results and discussion easier.

5. The calculation of economic costs needs to be described in more detail. The manuscript refers to opportunity costs, but it is not clear how these were measured or valued.

6. Were the same participants used in both study arms? If the populations differed, comparing the absolute number of TB cases detected may be misleading because the arm with more participants, or a population with a higher underlying TB risk, may naturally identify more cases. In addition to absolute numbers, the authors should present percentages, diagnostic yield, or cases detected per 1,000 people screened to allow a fairer comparison between the approaches.

Reviewer #2: General Summary and Recommendation

The manuscript describes a highly relevant, timely, and interesting study evaluating the economic and operational aspects of two active case finding (ACF) screening algorithms using AI (CAD4TBv7) and biomarker (POC-CRP) testing. The field-based data collected from South Africa and Lesotho provide crucial, real-world insights for National TB Programs balancing limited budgets. The paper is well-conceived; however, there are a few areas regarding textual clarity, methodological definitions, and the scope of the economic modelling that would benefit from further refinement. Therefore, I request the authors to address the recommendations detailed below before the manuscript is finalized for publication.

Detailed Comments for the Authors

1. Clarity and Structure of Screening Approaches (Lines 109–119)

• Please ensure the descriptions of the screening methodologies are presented with better consistency. When outlining the standalone CAD4TBv7 approach, the manuscript explains the outcomes for all participants (both below and above the threshold). However, the description of the combined CAD4TBv7-CRP approach only specifies the workflow for the intermediate and upper scoring tiers. The manuscript does not explicitly state the workflow or clinical outcome for participants falling into the lowest scoring tier (specifically, those scoring ≤5 in South Africa or ≤10 in Lesotho, as detailed in Supplementary Table 1). For completeness and to ensure parallel structure between both method descriptions, please explicitly state in the main text that these lower-tier participants were considered screen-negative.

• Additionally, replacing the generic term "window" with "triage window" would significantly improve readability and align the main text perfectly with the terminology used in the supplementary tables.

2. Choice of Denominator in the Full Analysis Set (FAS)

• While the justification for utilizing the Full Analysis Set for the cost analysis is understood, there appears to be a slight mathematical discrepancy. The authors report 72 TB cases for the CAD4TBv7 approach in the FAS, compared to 69 cases reported in the primary trial analysis.

• As the text notes, these three additional cases were excluded from the trial's primary analysis due to missing data in the alternative track. However, by including them in the denominator for the CAD-only cost-per-case calculation, the average cost per case in the CAD arm is mathematically reduced by approximately 4%.

• To ensure robust and conservative conclusions, it would be highly valuable if the authors could provide a sensitivity analysis showing the cost-per-case outcomes if the denominators were held strictly consistent with the primary trial outcomes (n=69 vs. n=60). This would clarify if this choice over-represented the cost-efficiency of the CAD-only approach.

3. Workforce Models and Task-Shifting Scenarios

• The cost analysis clearly demonstrates that personnel served as the dominant cost driver in South Africa, accounting for up to 58.8% of expenditures, and the average cost per participant screened was higher in South Africa than in Lesotho ($21 vs. $19).

• This pattern suggests that utilizing a senior nursing cadre in South Africa created a high fixed-cost burden that the program could not easily dilute, even with mobile deployment. To enhance the policy utility of the paper, would the authors consider modeling a hypothetical task-shifting scenario? Specifically, exploring whether adopting a junior nursing assistant model would alter the fiscal viability of the CAD4TBv7-CRP track in South Africa would add significant value.

4. Policy Implications and Sustainability of Vertical ACF

• The projections in Scenario 2 (scaling to 100,000 participants) present an important finding: the average cost per TB case detected did not significantly decrease with scale, primarily because recurrent costs (67–79%) scale proportionally with volume.

• Given that a flat economies-of-scale effect, it has major implications for National TB Programs, the authors are highly encouraged to expand their discussion on this point. It would be incredibly valuable to provide explicit policy guidance based on these data: at an observed prevalence of 0.3%, should vertical ACF programs be deprioritized in favor of integrated screening models, which successfully dropped the cost per case detected from $5,454 to $169?

5. Economic Performance of CRP in PLHIV Subpopulations

• The authors correctly highlight that the World Health Organization recommends point-of-care C-reactive protein testing specifically as a tuberculosis screening tool among people living with HIV. However, the results of this analysis indicate that the combined CAD4TBv7-CRP approach was 37% more expensive per case detected than the standalone CAD4TBv7 algorithm when deployed within the general community population.

• Given these findings, it is worth exploring whether the reported cost-inefficiency of the combined approach is driven by a "dilution effect" resulting from screening a broad, unselected population. If the economic evaluation were restricted exclusively to the subpopulation of participants living with HIV—the specific target demographic intended for this intervention—it is highly plausible that the cost-per-case detected would improve and potentially match or exceed the efficiency of the CAD-only track.

• Please provide a stratified sub-analysis or an expanded discussion comparing the cost of the CAD4TBv7-CRP approach within the PLHIV subgroup versus the general population. Incorporating this granular data is highly critical for informing National TB Programs, which may be evaluating the fiscal viability of implementing CRP triage within specialized HIV clinics rather than vertical, community-based active case-finding campaigns.

6. Clinical Utility and Impact of Xpert "Trace" Results

• The authors noted that active TB cases were defined by excluding Xpert "trace" results. However, in regions with a high burden of HIV co-infection like South Africa and Lesotho, trace results often carry significant clinical weight and frequently represent actionable, early-stage paucibacillary TB disease.

• To ensure the economic model captures the full clinical value of these digital screening tools, could the authors discuss or model how the inclusion of trace results in the "cases detected" denominator would impact the analysis? Clarifying whether this would narrow or widen the 37% cost disparity between the two algorithms would provide helpful context for clinicians and health economists alike.

Conclusion

This paper addresses a critical implementation gap in global health economics. Addressing these methodological recommendations and clarifying the text will elevate the manuscript to a highly robust piece of literature. I thank the authors for their valuable work and look forward to reviewing their updated submission.

**********

what does this mean?). If published, this will include your full peer review and any attached files.

**Do you want your identity to be public for this peer review?**  If you choose “no”, your identity will remain anonymous but your review may still be made public.

For information about this choice, including consent withdrawal, please see our Privacy Policy

Reviewer #1: No

Reviewer #2: **Yes:**  Suvesh Shrestha

**********

[NOTE: If reviewer comments were submitted as an attachment file, they will be attached to this email and accessible via the submission site. Please log into your account, locate the manuscript record, and check for the action link "View Attachments". If this link does not appear, there are no attachment files.]

# Author comment: Author response to Decision Letter 1

**DOI:** 10.1371/journal.pgph.0007341.r002

13 Aug 2026

Attachment Submitted filename: Response to reviewers.docx

# Peer review: Decision Letter 1

**DOI:** 10.1371/journal.pgph.0007341.r003

**Authors:** Rishi Gupta

1 Sep 2026

Comparative cost analysis of community-based tuberculosis screening: Computer-Aided Detection (CAD) alone versus CAD with point-of-care C-reactive protein testing in South Africa and Lesotho

PGPH-D-26-02457R1

Dear Mr Harkare,

We are pleased to inform you that your manuscript 'Comparative cost analysis of community-based tuberculosis screening: Computer-Aided Detection (CAD) alone versus CAD with point-of-care C-reactive protein testing in South Africa and Lesotho' has been provisionally accepted for publication in PLOS Global Public Health.

Before your manuscript can be formally accepted you will need to complete some formatting changes, which you will receive in a follow up email. A member of our team will be in touch with a set of requests.

Please note that your manuscript will not be scheduled for publication until you have made the required changes, so a swift response is appreciated.

IMPORTANT: The editorial review process is now complete. PLOS will only permit corrections to spelling, formatting or significant scientific errors from this point onwards. Requests for major changes, or any which affect the scientific understanding of your work, will cause delays to the publication date of your manuscript.

If your institution or institutions have a press office, please notify them about your upcoming paper to help maximize its impact. If they'll be preparing press materials, please inform our press team as soon as possible -- no later than 48 hours after receiving the formal acceptance. Your manuscript will remain under strict press embargo until 2 pm Eastern Time on the date of publication. For more information, please contact globalpubhealth@plos.org.

Thank you again for supporting Open Access publishing; we are looking forward to publishing your work in PLOS Global Public Health.

Best regards,

Rishi Kumar Gupta

Academic Editor

PLOS Global Public Health
